# The *hedgehog* Pathway Gene *shifted* Functions together with the *hmgcr*-Dependent Isoprenoid Biosynthetic Pathway to Orchestrate Germ Cell Migration

**DOI:** 10.1371/journal.pgen.1003720

**Published:** 2013-09-12

**Authors:** Girish Deshpande, Keren Zhou, Joy Y. Wan, Jana Friedrich, Nicholas Jourjine, Daniel Smith, Paul Schedl

**Affiliations:** 1 Department of Molecular Biology, Princeton University, Princeton, New Jersey, United States of America; 2 Centre for Organismal Studies (COS) University of Heidelberg, Heidelberg, Germany; 3 Institute of Gene Biology RAS, Moscow, Russia; Stanford University School of Medicine, United States of America

## Abstract

The *Drosophila* embryonic gonad is assembled from two distinct cell types, the Primordial Germ Cells (PGCs) and the Somatic Gonadal Precursor cells (SGPs). The PGCs form at the posterior of blastoderm stage embryos and are subsequently carried inside the embryo during gastrulation. To reach the SGPs, the PGCs must traverse the midgut wall and then migrate through the mesoderm. A combination of local repulsive cues and attractive signals emanating from the SGPs guide migration. We have investigated the role of the *hedgehog* (*hh*) pathway gene *shifted* (*shf*) in directing PGC migration. *shf* encodes a secreted protein that facilitates the long distance transmission of Hh through the proteoglycan matrix after it is released from basolateral membranes of Hh expressing cells in the wing imaginal disc. *shf* is expressed in the gonadal mesoderm, and loss- and gain-of-function experiments demonstrate that it is required for PGC migration. Previous studies have established that the *hmgcr*-dependent isoprenoid biosynthetic pathway plays a pivotal role in generating the PGC attractant both by the SGPs and by other tissues when *hmgcr* is ectopically expressed. We show that production of this PGC attractant depends upon *shf* as well as a second *hh* pathway gene *gγ1*. Further linking the PGC attractant to Hh, we present evidence indicating that ectopic expression of *hmgcr* in the nervous system promotes the release/transmission of the Hh ligand from these cells into and through the underlying mesodermal cell layer, where Hh can contact migrating PGCs. Finally, potentiation of Hh by *hmgcr* appears to depend upon cholesterol modification.

## Author Summary

The molecular mechanisms underlying directed cell migration have been studied extensively in different biological contexts. Germ cell migration provides an effective model to study the dynamics of in vivo cell migration. The process of germ cell migration in *Drosophila melanogaster* results in embryonic gonad formation consisting of primordial germ cells (PGCs) and somatic gonadal precursor cells (SGPs). Moreover, it likely involves a complex series of attractive and repulsive cues. Molecular and genetic analysis has been performed to elucidate the nature of the attractive cue(s) and components that guide germ cells to the SGPs in the mesoderm. One current model proposes that 3-Hydroxy-3-Methylglutaryl Coenzyme A reductase (Hmgcr), synthesized in the SGPs, potentiates signaling downstream of Hedgehog (Hh) ligand also emanating from the SGPs. The model pivots on the novel activity of an established morphogen, Hedgehog, to function as a chemoattractant for the migrating germ cells. A variety of ‘loss-’ and ‘gain-of-function’ strategies manipulating different components of this signaling pathway have been successfully employed in support of the proposed model.

## Introduction

The *hedgehog* (*hh*) signaling pathway plays a crucial role in patterning in a wide range of multicellular eukaryotes [1]–[4]. In addition to its role in morphogenesis, *hh* has also been implicated in cell migration, axonal guidance and in shaping polarized cellular extensions [5]–[9]. In most of these contexts, Hh must be able to signal not only to nearby cells but also to cells located at a distance. Recent studies have uncovered an unusual mechanism for long distance *hh* signaling.

The Hh ligand in *Drosophila* is synthesized as a precursor polypeptide, which is subject to a series of processing steps [10],[11]. It is initially targeted to the secretory pathway by an N-terminal signal sequence. After removal of the signal sequence, an internal autoproteolytic cleavage coupled with cholesterol addition generates the active 19 kD, Hh-Np (processed) ligand. This peptide is then palmitoylated before it is released from the apical surface of polarized epithelial cells. Although Hh-Np can signal to neighboring cells, its movement along the apical surface is constrained by the lipid modifications, in part through interactions with the glypican Dally, and the transmembrane protein Brother of Ihog [12]–[14]. By contrast, a C-terminal truncation, Hh-N, that lacks the cholesterol modification, diffuses much farther along the apical surface than Hh-Np [15]. Additionally, movement of the unmodified Hh-N ligand is independent of factors like *tout-velu* (*ttv*) and *dispatched* (*disp*) that are critical for the spreading of the fully modified Hh-Np protein [16]–[19].

Recent studies by Callejo et al. [20] have suggested that long distance *hh* signaling is mediated by an unusual multistep pathway in which Hh-Np is transmitted from the basolateral membranes of the sending cells. In this model, most of the Hh-Np that reaches the apical surface is recaptured and internalized by an endocytic pathway that depends upon dynamin and Rab5. Instead of being targeted for lysosomal degradation, the Hh-Np endosomes are recycled by a Rab8 dependent mechanism into exocytic vesicles that are targeted to the basolateral membrane of the *hh* expressing cells. It is possible that Disp, with its sterol sensing domain, plays a role in selecting Hh-Np containing endocytic vesicles for recycling to the basolateral membrane. Disp is found co-localized with Hh-Np in endocytic vesicles near the apical surface and along the basolateral membrane. It is also in an immunoprecipitable complex with Hh-Np. Two proteins previously implicated in facilitating the response of cells receiving the Hh signal, the Dally-like protein (Dlp) and Interference Hh (Ihog), also seem to participate in the targeting and/or release of Hh-Np from the basolateral membrane.

Other proteins have been implicated in the basolateral release and transmission of Hh-Np from *hh* expressing cells [10], [21], [22]. One of these, HMGCoA reductase (*hmgcr*), contains a transmembrane sterol-sensing domain like *disp*. HMGCoA reductase is responsible for the synthesis of mevalonic acid, which is a precursor for isoprenoid and sterol biosynthesis, and is a key regulatory target in both biosynthetic pathways [23]. In *hmgcr^−^* mutant embryos, Hh-Np is not properly released from sending cells and instead accumulates in punctate aggregates along the basolateral membranes. Unlike vertebrates, flies are unable to synthesize cholesterol from mevalonic acid [24], and Hh is modified by exogenous cholesterol. However, mevalonic acid is nevertheless required for *hh* signaling because it is a precursor for the synthesis of the isoprenoid geranylgeranyl-pyrophosphate by geranylgeranyl diphosphate synthetase (*qm*). Like *hmgcr*, *qm* mutations impede the release of Hh-Np from *hh* expressing cells [22]. Geranylgeranyl-pyrophosphate is ultimately used in protein geranylation. In *hh* signaling, a key target for geranylation is the G protein γ subunit 1 (Gγ1) [22]. Gγ1 is a subunit of the heterotrimeric GαGβGγ1 complex which helps mediate the intracellular trafficking of membrane vesicles and cargo [25], [26]. To be active, this heterotrimeric complex must be anchored to the membrane by Gγ1 geranylation.

Many of the factors implicated in the basolateral release and subsequent transmission of Hh-Np in polarized epithelium also function in the signaling pathway(s) that directs migration of primordial germ cells (PGCs) to the somatic gonadal precursor cells (SGPs) during mid-embryogenesis [8], [21], [22], [24], [27]. The PGCs form at the posterior pole of the embryo during the syncytial blastoderm stage, while the SGPs arise during mid-embryogenesis from the lateral mesoderm in parasegments 10–13. In order to coalesce with the SGPs and form the embryonic gonad, the PGCs must traverse from the posterior end into the middle of the embryo and then subsequently move to the lateral mesodermal cell layer, which harbors the newly formed SGPs [28].

While it is generally agreed that an attractive signal(s) produced by the SGPs is needed to guide PGC migration, the identity of the attractant(s) and how *hh* or other *hh* pathway genes like *hmgcr*, *qm* and *gγ1* function in migration remains unresolved and controversial [8], [28]–[30]. To further elucidate the role of this signaling pathway in PGC migration, we have examined the *shifted* (*shf*) gene. *shf* encodes an extracellular protein that is the *Drosophila* ortholog of the vertebrate Wnt Inhibitory Factor-1 (WIF-1) [13], [14], [31]–[33]. In vertebrates Wif1 antagonizes *wnt* signaling by capturing Wnt and anchoring it to the heparan sulfate proteoglycan [31], [34]. Unlike its vertebrate counterpart, Shf has no role in Wnt signaling. Instead, it functions in the *hh* pathway. The Shf protein is enriched in the basolateral ECM, and it facilitates the long distance transmission of Hh-Np after it has been released from the basolateral membranes of signaling cells. In *shf* mutants, the basolateral accumulation and subsequent spreading of Hh-Np in the wing disc is disrupted, while apical accumulation appears to be relatively normal. These defects in Hh-Np transmission result in a reduction in the expression of *hh* targets in the anterior compartment of the wing.

Guerrero and colleagues [35] [Guerrero pers. comm.] have found that the defects in *hh* signaling in the wings of *shf* mutants can be suppressed by overexpression of *hmgcr*. Since *hmgcr* plays a pivotal role in generating the PGC attractant [27], [29], [31], an intriguing possibility is that the functional connection to *shf* seen in the wing could also be of significance in PGC migration. Here we have first tested whether *shf* functions in PGC migration using both ‘loss’ and ‘gain’ of function strategies. We have then used genetic ‘epistasis’ experiments to link *shf* and the *hh* pathway gene *gγ1* to the functioning of the *hmgcr→qm* isoprenoid biosynthetic pathway in PGC migration. Finally we use co-expression experiments to show that *hmgcr* potentiates the transmission of *hh-GFP* out of the nervous system and into the mesoderm, where it associates with PGCs.

## Results

### Increase in Shf levels in the sending cells is sufficient to enhance *hh* target gene expression

**Figure 1:**
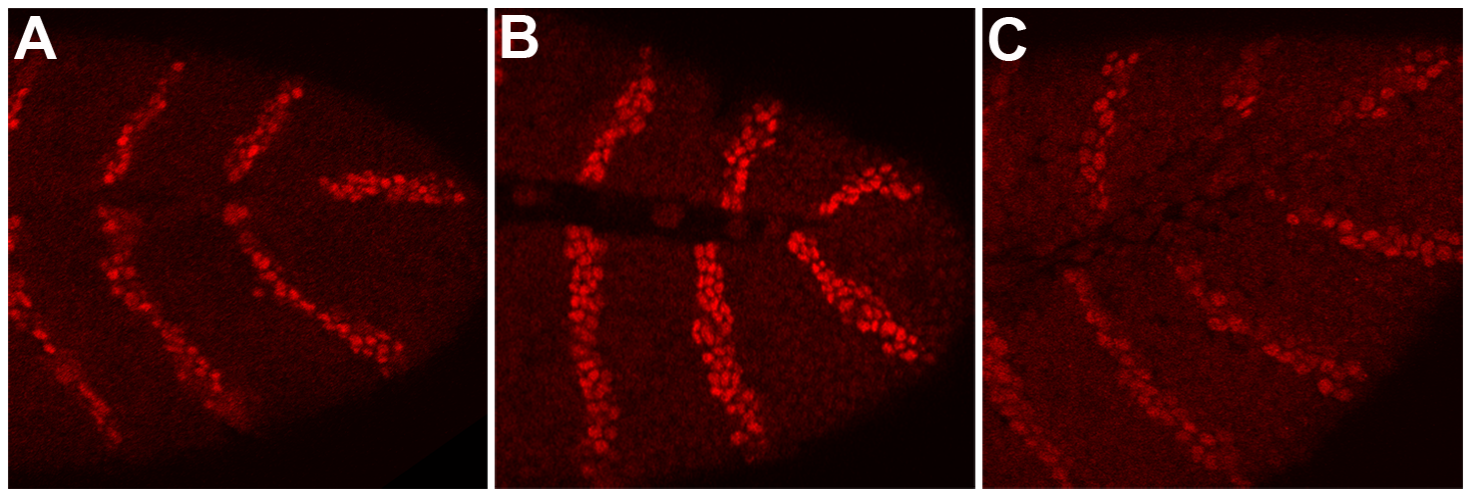
Overexpression of *shf* upregulates the *hh→wg*→*en* autoregulatory loop. Embryos were generated by mating females homozygous for the *UAS-shf* transgene with *hh-GAL4*/TM6 *Ubx-LacZ* or *ptc GAL4/ptc-GAL4* males. The processed embryos were stained with Engrailed (En) or En and β-galactosidase antibodies. (A) *ptc-GAL4/UAS-shf*, (B) *hh-GAL4/UAS-shf* (C) control *UAS-shf/Ubx-LacZ*. It is worth noting that Shf is a secreted protein, and studies on the wing disc indicate that it can rescue the *shf* mutant phenotype when ectopically expressed in either *hh* sending or *hh* receiving cells [30]. In this respect, *shf* functioning in the embryo would seem to be somewhat different in that it appears to be more effective in potentiating *hh* signaling when expressed in *hh* sending than in receiving cells. On the other hand, this difference may simply reflect the fact that *shf* is expressed in a stripe pattern in the embryonic ectoderm (see Figure 2).

Even though *shf* is expressed during embryogenesis, *shf* mutant embryos have no discernable patterning defects. The likely reason for this is that Hh-Np needs to spread only 3–4 cell diameters in the embryonic ectoderm [11], [12], [36] whereas a much larger distance (12–13 cell diameters) is required for patterning the wing imaginal disc [11], [15], [20], [32], [33]. To confirm that *shf* is nevertheless able to promote *hh* signaling during embryogenesis we asked if ectopic *shf* can upregulate the *hh→wingless* (*wg*)→*engrailed* (*en*) autoregulatory loop. In this loop, *hh* expressing cells in each parasegment signal to the neighboring anterior cells to activate *wingless* (*wg*) expression. Wg in turn signals back to the row of *hh* expressing cells and upregulates *en* expression. To direct *shf* expression in *hh* sending cells we used an *hh-Gal4* driver, while we used a *patched-Gal4* (*ptc-Gal4*) driver to express *shf* in *hh receiving* (*wg* expressing) cells. Figure 1 shows that the level of En protein in the stripes is appreciably increased over that seen in wild type stripes (panel C) when excess Shf is produced using the *hh-Gal4* driver (panel B). While driving Shf in *wg* expressing cells using *ptc-Gal4* also appears to upregulate the En stripes, the increase over wild type is less pronounced than with the *hh-Gal4* driver. To confirm that *shf* can potentiate *hh* signaling in the embryo we examined the accumulation of Wg in *hh-Gal4:UAS-shf* embryos. Figure S1 shows that Wg accumulation in *hh* receiving cells is upregulated by expressing *shf* in *hh* sending cells.

### *shifted* is expressed in the mesoderm

**Figure 2:**
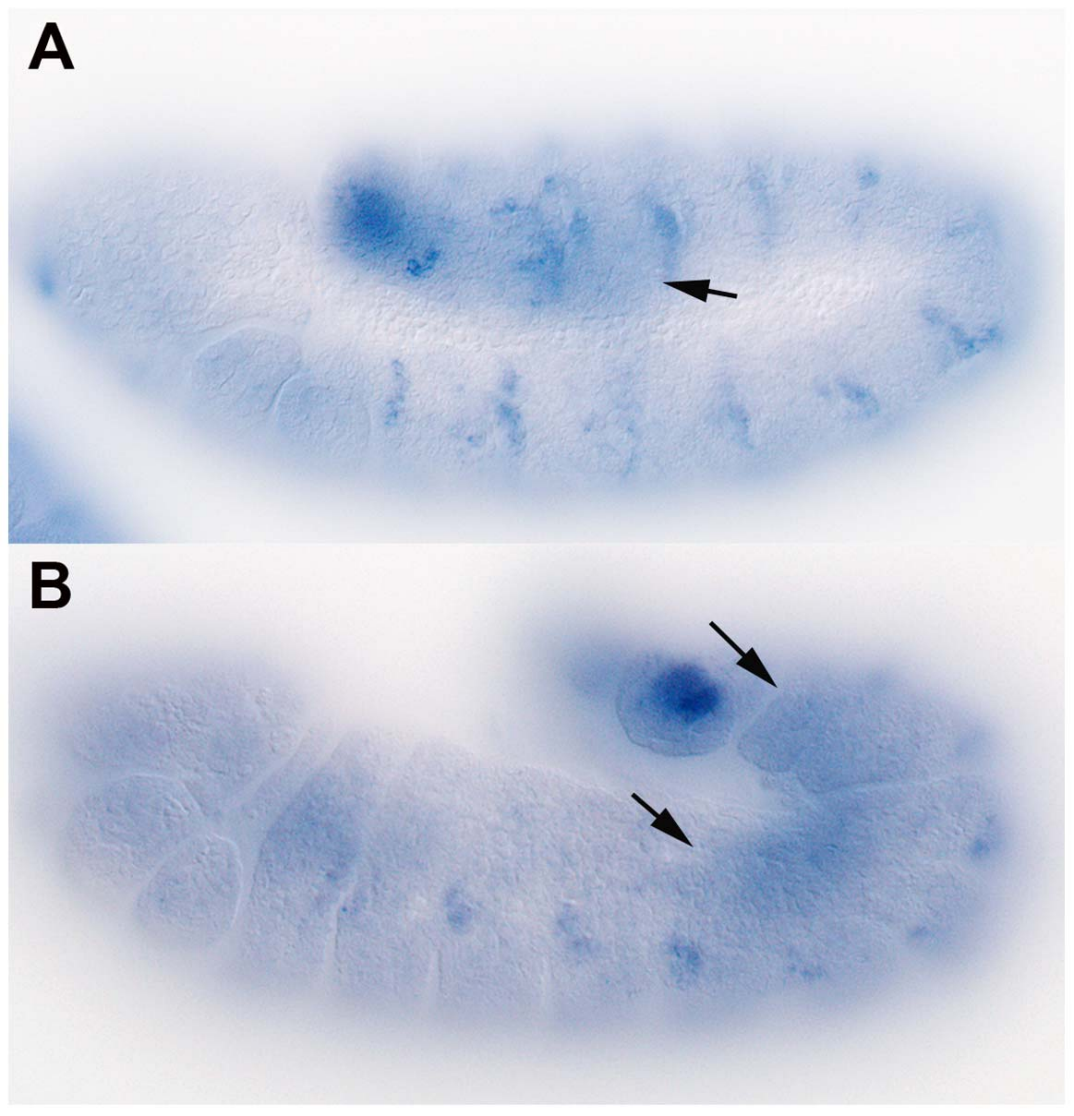
*shf* is expressed in the embryonic mesoderm. Whole-mount *in situ* hybridization was performed on staged wild type embryos using anti-sense RNA probes for *shf* (GH27042) mRNA. A: Stage 11 embryo showing segmental distribution of *shf* mRNA in the ectoderm. B: Late stage 12 embryo showing that *shf* mRNA is enriched in the mesoderm in parasegments 10–13. Arrows indicate mesoderm expression.

While long distance signaling might not be essential for patterning of the embryonic ectoderm, attractants produced by the SGPs must travel from the posterior mesoderm to the midgut in order to direct migrating PGCs. If Hh-Np functions as a PGC attractant, then *shf* could have a role in PGC migration, in which case it should be expressed in the mesoderm. Previous studies indicate that *shf* mRNA is maternally deposited and is uniformly distributed in blastoderm stage embryos [32], [33]. By mid-embryogenesis, *shf* mRNA is expressed in a segmentally repeating pattern in the ectoderm. This is shown in the stage 10 embryo in Figure 2A. *shf* expression in the mesoderm (arrow) can also be detected at the posterior. The mesodermal expression is shown more clearly in the stage 12–13 embryo in panel B. Expression in the mesoderm appears to be highest in segments A4–A8, which is the region of the embryonic mesoderm that gives rise to the SGPs, while it is lower in the more anterior segments, which give rise to fat body cells. The upregulation of *shf* expression in more posterior segments would fit with recent studies of Zhai et al. [37], who showed that *shf* is regulated by the Bithorax complex gene, *Abdominal-B*. It should be noted that though *shf* seems to be preferentially expressed in the posterior mesoderm during the period when PGCs are migrating, its pattern of expression is much broader than *hmgcr*, which at this stage is only expressed in a subset of mesodermal cells, the SGPs [27].

### Embryos compromised for *shifted* function display germ cell migration defects

We next asked whether *shf* is needed for proper PGC migration. For this purpose we examined three different *shf* alleles, *shf^2^*, *shf^x33^* and *shf^EY03173^*. *shf^2^* has a missense mutation in the third EGF repeat, replacing a conserved cysteine (Cys374) with a serine. *shf^2^* flies are viable and fertile; however, their wings display the typical *shf* wing phenotype (i.e. a narrowing of the L3–L4 intervein space) [32], [33]. The *shf^x33^* mutant has a 630 bp deletion and is considered a null allele [30]. Male *shf^x33^* flies show slightly stronger *shf* wing and eye phenotypes than *shf^2^*. Finally *shf^EY03173^* is a P-element insertion into the middle of the *shf* transcription unit. It exhibits the *shf* wing and eye phenotypes and is semi-lethal.

**Figure 3:**
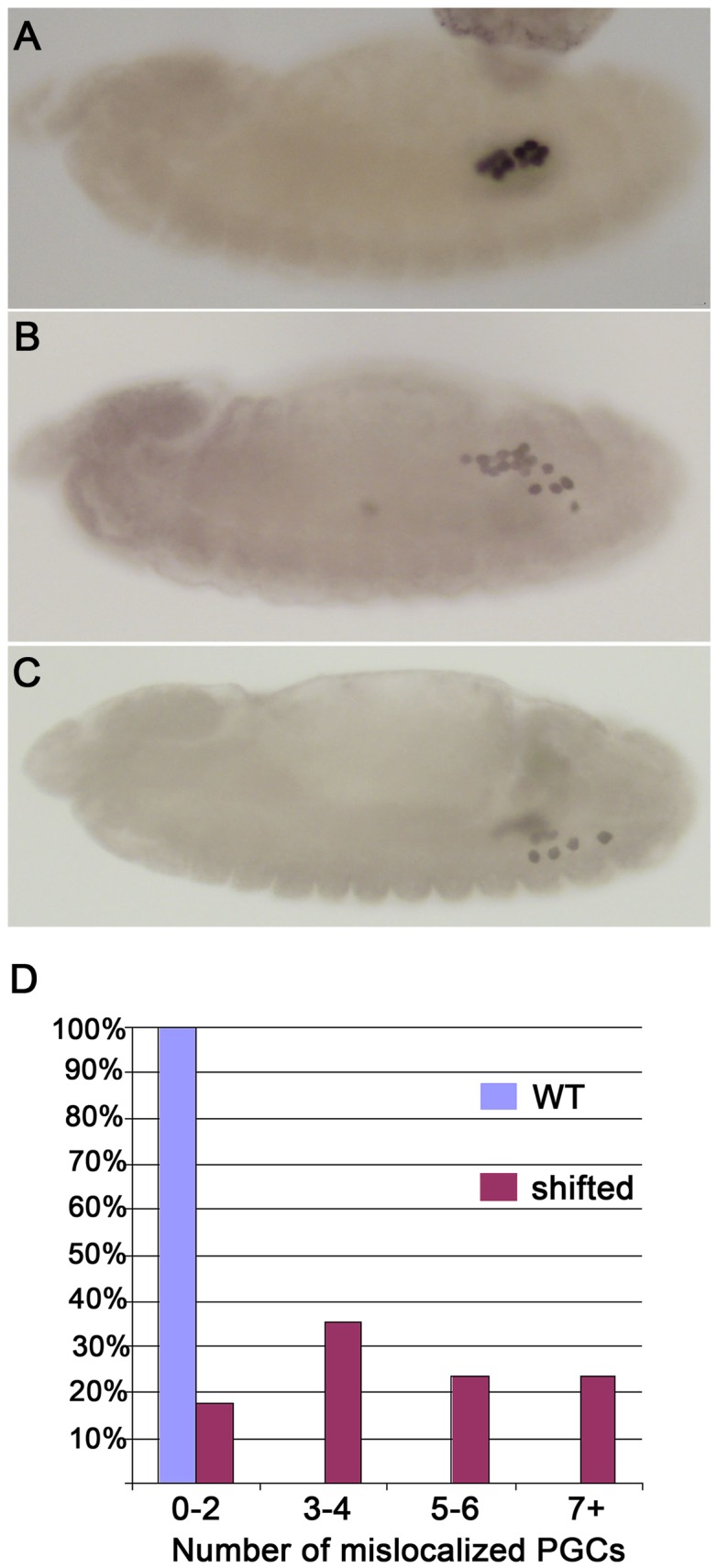
*shf* is required for proper PGC migration. Embryos were generated by mating *shf^x33^/FM7 ftzβ–gal* females to *shf^2^* males. PGCs were identified using Vasa antibody, while β-gal antibody was used to identify balancer males and females. The *shf* embryos shown in B–D are from the cross. (A): Wild type stage 13 embryo with PGCs and SGPs properly aligned. (B) and (C): Stage 13 and stage 14 *shf^−^*embryos, respectively. Many of the PGCs are scattered instead of being aligned with the SGPs in the stage 13 embryo and then don't coalesce into the embryonic gonad at stage 14. (D) Plots of the number of scattered or mislocalized PGCs in wild type and *shf trans*-heterozygous embryos. Wild type: N = 20; *shf*: N = 17. The embryos were classified as indicated into four different categories based on number of mislocalized PGCs. Migration defects were also observed in *shf^2^* and *shf^EY03173^* embryos (not shown).

We found that the early steps in PGC development from their formation through stage 12 are unaltered in *shf* embryos. However, as shown in Figure 3 for the *trans*-heterozygous *shf^2^*/*shf^x33^* combination, migration phenotypes are evident by the end of stage 13. At this point in wild type embryos, all of the PGCs have migrated from the midgut to the mesoderm and are aligned with the SGPs. By comparison, in *shf* mutants, a subset of the PGCs lingers behind on the outside surface of the midgut. Interestingly, a similar though more pronounced lingering phenotype is observed in *hmgcr* embryos. By the time of gonad covalence, we find that over 40% of the *shf* embryos have 5 or more PGCs scattered in the posterior as compared to two or less for wild type (Figure 3). Similar results were obtained for individual *shf* mutants (not shown).

### Ectopic expression of *shifted* disrupts germ cell migration

To further substantiate a role for *shf* in PGC migration, we tested the effects of ectopic expression. Previous studies have shown that misexpression of genes that clearly cannot encode the attractant itself (but instead code for products involved in its production, release or transmission: *hmgcr*, *qm*, *farnesyl-diphosphate synthase* (*fpps*) and *gγ1*) can disrupt the migration of PGCs towards the SGPs [22], [24], [27]. Even more remarkable, these genes can be expressed in many unrelated tissues and cell types, yet are still capable of generating an attractant that can compete with the one produced by the SGPs.

**Figure 4:**
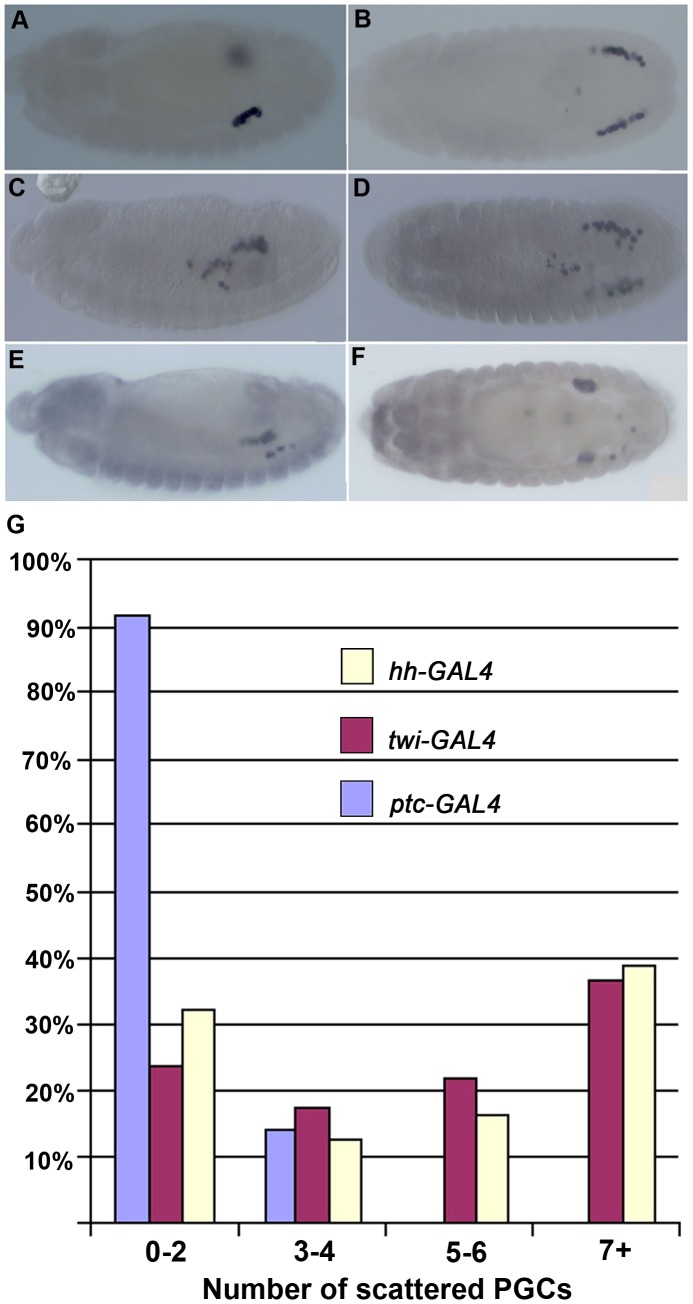
Ectopic *shf* expression induces PGC migration defects. Images in A–F show stage 13–14 (A, B) *ptc-GAL4/UAS-shf* (C, D), *twi-GAL4/UAS-shf* and (E, F) *hh-GAL4/UAS-shf* embryos probed with Vasa antibody to identify PGCs. Only very minor PGC migration defects are evident in *ptc-GAL4/UAS-shf*, while many scattered PGCs are seen with the *twi* and *hh* drivers. Panel at the bottom shows quantitation of the PGC migration defects observed with the 3 different drivers as indicated. The frequency of PGC migration defects typically seen in wild type controls (lacking the *GAL4* driver and the *UAS-shf* transgene) are shown in Figure 3. Blue *ptc-GAL4/UAS-shf* N = 47. Red *twi-GAL4/UAS-shf* N = 68. Yellow *hh-GAL4/UAS-shf* N = 31. D) Plots of the number of scattered or mislocalized PGCs when *shf* was ectopically expressed with the driver as indicated in the graph.

To determine if *shf* also belongs to this special group of genes, we used *hh-GAL4* and *ptc-GAL4* drivers to ectopically express *shf*. Since *shf* ectopic expression using the *hh-GAL4* driver was more effective in promoting *hh* signaling in the embryonic ectoderm than the *ptc-GAL4* driver, we anticipated that any effects on PGC migration would likely be stronger with *hh-GAL4*. Figure 4 shows that this is the case. While about 15% of the *ptc-GAL4:UAS-shf* embryos had three to four mislocalized PGC, the vast majority resembled wild type (Figure 4A & B). By contrast, nearly 40% of the *hh-GAL4:UAS-shf* embryos had seven or more mislocalized PGCs (Figure 4E & F).

### Germ cell migration is sensitive to ubiquitous *shifted* expression in the mesoderm

We sought to further confirm the effects of ectopic *shf* on PGC migration and to compare *shf* with other genes implicated in this process. For ectopic expression we used the pan-mesodermal driver *twist-GAL4*. For the purposes of comparison, we selected *hmgcr*, *qm*, *gγ1*, *ttv*, and *hh*. As described above, the *hmgcr→qm→gγ1* pathway functions in the release of the Hh-Np ligand from sending cells, while *ttv* provides the proteoglycan matrix that sustains and promotes its transmission.

**Figure 5:**
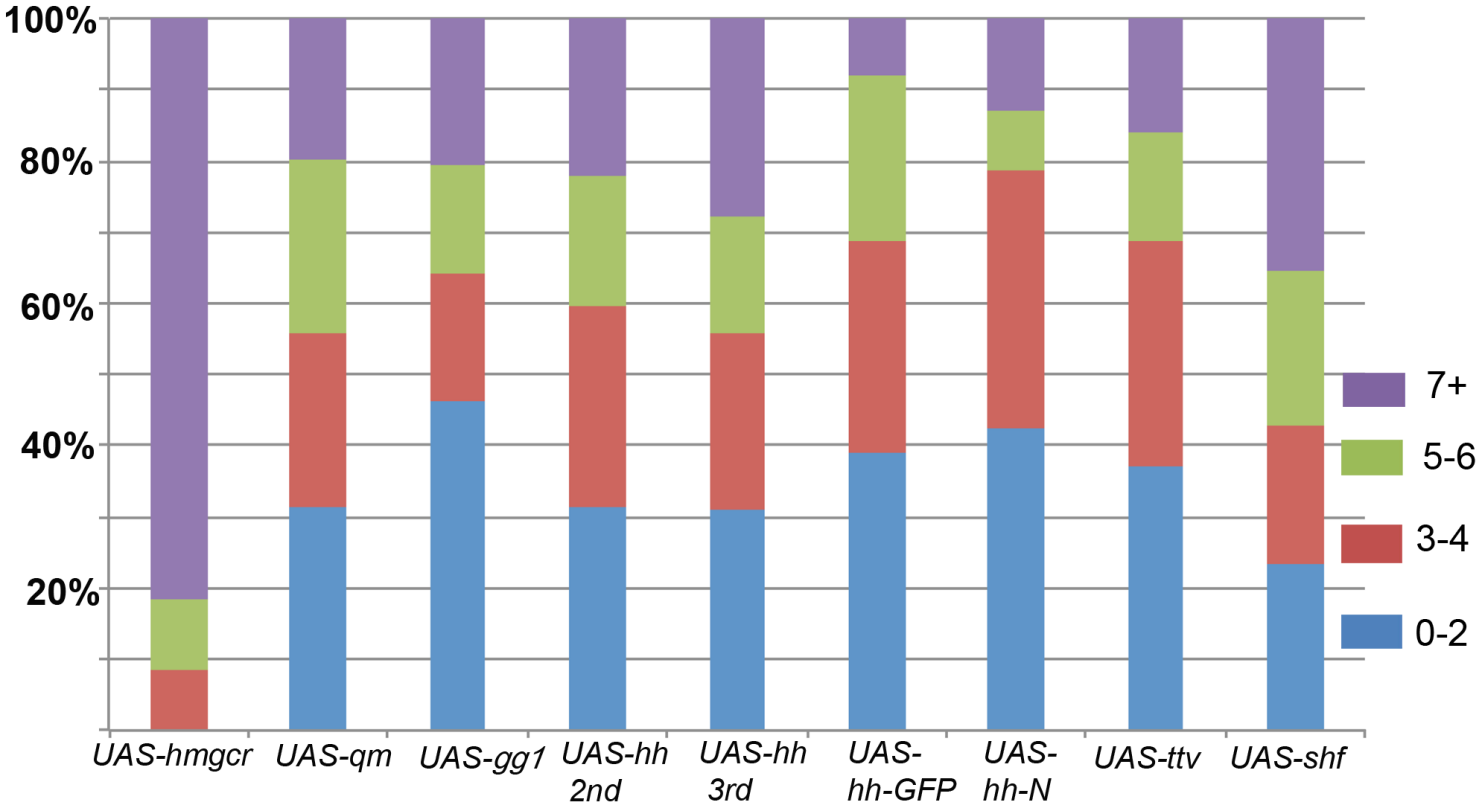
Migration defects induced by ectopic expression of *hh* signaling pathway genes in the mesoderm. Homozygous *twi-GAL4* females were mated with males homozygous for the following *UAS* transgenes: *hmgcr*, *qm*, *gγ1*, *hh* (on 2^nd^ and 3^rd^ chromosomes), *hh-GFP*, *ttv*, and *shf*. Purple: 7+ scattered PGCs; Green: 5–6 scattered PGCs; Red: 3–4 scattered PGCs; Blue: 0–2 scattered PGCs. *hmgcr:* N = 63; *qm*: N = 86; *gγ1*: N = 78; *hh 2^nd^*: N = 131; *hh 3^rd^:* N = 152; *hh-GFP*: N = 64; *UAS-hh-N* (3^rd^ chromosome): N = 47); *ttv*: N = 132; *shf*: N = 68. Number of scattered PGCs in wild type embryos is shown in Figure 3. Bars show percentage of embryos in each category as indicated.

Previous studies have shown that amongst the genes known to function in germ cell migration *hmgcr* is a pivotal player in that it encodes a limiting factor for generating an ectopic attractant that can misdirect PGCs. This is illustrated by the dramatic effects of *hmgcr* misexpression in the mesoderm shown in Figure 5. Over 90% of the embryos have 5 or more mismigrated PGCs, while more than 80% had 7 or more mismigrated PGCs. Similar results have been reported for misexpression in the nervous system ([27]: see below).

At least part of the explanation for the unusual potency of *hmgcr* is that its expression is restricted to the SGPs at the time the PGCs commence their migration. By contrast, all of the other known genes, with the exception of the mesoderm specific MDR49 [38], are more widely expressed in the embryo. Thus, there may only be an incremental increase in the production or transmission of an ectopic PGC attractant when these genes are overexpressed in tissues in which they are already present. With the important caveat that the levels of gene product generated by different *UAS* transgenes may not be equivalent, Figure 5 shows that while ectopic expression of *qm*, *gγ1*, and *ttv* perturbs PGC migration, their effects are appreciably less severe than *hmgcr*. For example, whereas over 90% of the *twi-GAL4/UAS-hmgcr* embryos had 5 or more scattered PGCs, only about 30% of the *twi-GAL4/UAS-ttv* embryos had 5 or more scattered PGCs. Though ectopic *shf* causes greater disruptions in PGC migration than some of these other genes, its effects are still less than *hmgcr*.

Consistent with the results of previous studies [8], ectopic expression of the putative PGC attractant, *hh*, is also less effective than *hmgcr*. With two different transgenes encoding wild type Hh-Np, we found that about 40% of the embryos had 5 or more scattered PGCs, while about 25% had 7 or more scattered PGCs. (The effects of *hh* misexpression on PGC migration are considered further in Text S1.) We also tested transgenes expressing an Hh-GFP fusion protein and the C-terminal truncation Hh-N. The Hh-GFP fusion protein is thought to be processed and transmitted much like the wild type Hh-Np; however, this particular transgene doesn't fully rescue a *hh* temperature mutant [39]. As shown in Figure 5, the *hh-GFP* transgene is less effective than either of the *hh-Np* transgenes. The same is true for a transgene expressing the truncated Hh-N protein, which lacks the C-terminal cholesterol modification.

### *shifted* and *gγ1* mutations suppress PGC migration defects induced by ectopic *hmgcr*

The PGC attractant generated by expression of *hmgcr* or other genes at ectopic sites is thought to be the same as the attractant produced by these same genes in the SGPs [28], [30]. This means that the synthesis, release and long distance transmission of the ectopically produced attractant should in most instances depend upon precisely the same set of genes and pathways as those involved in generating the bona fide SGP attractant. In principle, it should be possible to exploit this co-dependence to explore the relationship between the different genes involved in generating the attractant. For these experiments we focused on *hmgcr*, as it plays such a central role in signaling the migrating PGCs [27]. We used an *elav* driver to express *hmgcr* in the nervous system.

**Figure 6:**
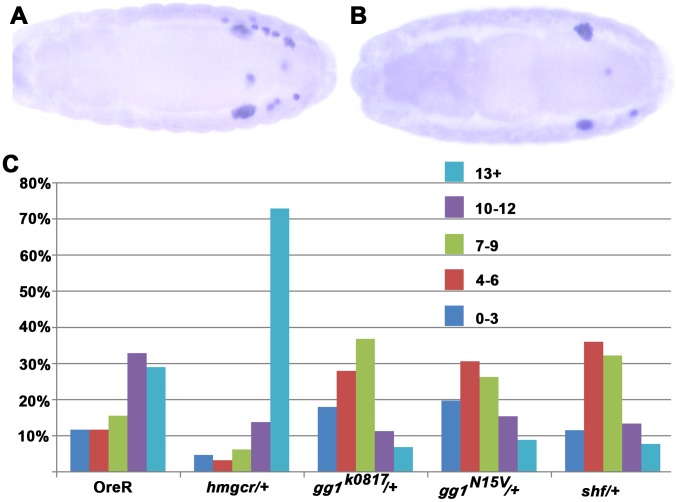
Mutations in *gγ1* and *shf* dominantly suppress the PGC migration defects induced by ectopic expression of *hmgcr* in the nervous system. Panel A: Hemizygous stage 15 *elav-GAL4*, *UAS-hmgcr/+* embryo. Panel B: Stage 15 *trans-*heterozygous *elav-GAL4*, *UAS- hmgcr/gγ1^k0817^* embryo. C. Quantitation of the PGC migration (visualized with Vasa antibody) defects in embryos hemizygous for the *elav-GAL4*, *UAS-hmgcr* recombinant chromosome and heterozygous for mutations in *hmgcr*, *gγ1*, and *shf*. OreR: Homozygous *elav-GAL4*, *UAS- hmgcr* males were crossed to wild type females (OreR). *hmgcr/+*: Homzygous *elav-GAL4*, *UAS- hmgcr* males were crossed to *hmgcr* ^1^/*TM3 Sb Ubx-lacZ* females. Embryos heterozygous for *hmgcr* ^1^ were identified as β-galactosidase negative. *gγ1/+*: Homozygous *elav-GAL4*, *UAS- hmgcr* males were crossed to the indicated *gγ1/CyO Ubx-lacZ* mutant. Embryos heterzygous for *gγ1* were identifed as β-galactosidase negative. *shf/*+: Homzygous *shf* ^112^ females were mated with homozygous *elav-GAL4 UAS hmgcr* males. Female embryos heterozygous for the *shf* mutation were identified as Sxl positive and scored. Male embryos were not scored.

In order to misdirect migrating PGCs, the attractant generated in the nervous system by ectopic *hmgcr* must compete with the attractant produced by the SGPs. Thus, reducing the gene dose of a factor that is more critical for the activity of the SGP derived attractant than it is for the nervous system derived attractant should exacerbate the effects of ectopic *hmgcr*. One example of such a factor would be *hmgcr* itself. Since expression of the endogenous gene is restricted to the SGPs during PGC migration, reducing the *hmgcr* gene dose in the nervous system should have little or no effect on the competitive activity of the ectopic attractant. In contrast, production of the attractant by the SGPs should be diminished when there is only a single wild type copy of *hmgcr*, making it easier for the ectopic nervous system signal to misdirect the migrating PGCs. Figure 6 shows that this prediction is correct—reducing *hmgcr* gene dose leads to an increase in the number of scattered PGCs. Similar effects would be predicted for other genes that are expressed or function only in SGPs (or mesoderm) but are not expressed and/or not required in the nervous system.

For signaling genes whose expression is not restricted to the SGPs or the mesoderm, reducing their dose could, like *hmgcr*, have a greater effect on the attractant from the SGPs than on the ectopic attractant. However, it also possible that the factors encoded by these genes might be more limiting for the production or transmission of the ectopic attractant in the nervous system than they are in the SGPs. In this case, reducing their dose should suppress rather than enhance the effects of ectopic *hmgcr* on PGC migration. For these experiments we selected *shf* and a second gene *gγ1* whose relationship to *hmgcr* is well defined. The Gγ1 protein must be geranylated in order to be active, and in *hh* signaling it is an important target for the *hmgcr→fpps→qm* isoprenoid biosynthetic pathway. Figure 6 shows that the migration defects induced by ectopic *hmgcr* are mitigated when the embryos are heterozygous for two different *gγ1* alleles. Whereas about 60% of the *elav-GAL4,UAS-hmgcr* embryos have 10 or more scattered PGCs, this number drops to less than 25% for both *gγ1* alleles. This finding would argue that *gγ1* must also function downstream of *hmgcr* in the production or transmission of the ectopic attractant. This conclusion would fit with previous studies which showed that ectopic expression of a dominant negative Gγ1 protein that can't be geranylated and poisons the heterotrimeric GαGβGγ1 complex disrupts PGC migration. We next tested *shf*. Like *gγ1* the number of embryos with 10 or more scattered PGCs is reduced, going from 60% to 20%. This finding would argue that production or transmission of the attractant induced at ectopic sites by *hmgcr* expression depends upon a second *hh* signaling gene *shf*.

### *hmgcr* induced migration defects are enhanced by expressing *hh* pathway genes

**Figure 7:**
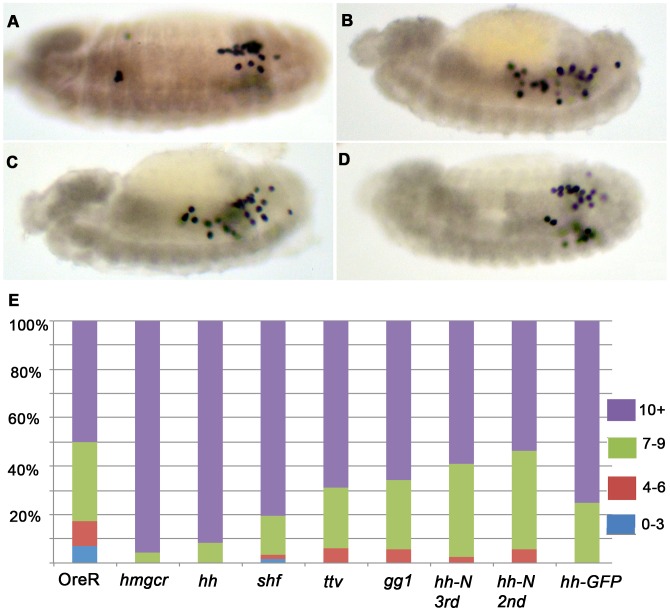
Enhancement of the *elav*-*GAL4 UAS*-*hmgcr* PGC migration defects by co-expression of *hh* pathway genes. Panels A–D. Homozygous *elav-GAL4 UAS-hmgcr* females were crossed with males homozygous for different transgenes as follows: (A) OreR (wild-type/no transgene), (B) *UAS-hmgcr*, (C) *UAS-hh*, and (D) *UAS-shf*. The embryos shown in (A–D) are at stage 13. Panel E) The bar graph at the bottom of the figure shows the frequency of embryos having different numbers of scattered PGCs in embryos hemizygous for both the recombinant *elav-GAL4*, *UAS-hmgcr* chromosome and one of the UAS transgenes (except OreR) as indicated. Ore-R (N = 58); *UAS*-*hmgcr* (N = 92); *UAS-hh* (N = 96); *UAS-shifted* (N = 62); *UAS-ttv* (N = 32); *UAS-gγ1* (N = 35); UAS-*hh-N* (3^rd^ chromosome) (N = 39); *UAS-hh-N* (2^nd^ chromosome) (N = 54); *UAS-hh-GFP* (N = 60).

To further explore the functional connections between *hmgcr* and *hh* pathway genes in the production of an ectopic PGC attractant in the nervous system, we examined the effects of co-expression. For this purpose females carrying a recombinant chromosome containing both *elav-GAL4* and *UAS-hmgcr* were mated to males carrying *UAS* transgenes for *shf*, *ttv* and *gγ1*. As a positive control we used *UAS-hmgcr*, as we already had evidence that its effects on germ cell migration were dose dependent. Figure 7A provides a further demonstration that the limiting factor in generating an ectopic PGC attractant is *hmgcr*. Adding a second copy of *UAS-hmgcr* essentially eliminates the intermediate migration phenotypes, and almost all embryos have greater than 10 scattered PGCs. Figure 7E shows that co-expressing *ttv*, *gγ1* and *shf* in the nervous system also exacerbates the effects of ectopic *hmgcr*; however, none is equivalent to an extra copy of *hmgcr*.

### *hh* but not *hh-N UAS*-transgenes enhance the *hmgcr* induced migration defects

We next co-expressed *hmgcr* with wild type Hh-Np or the C-terminal truncation, Hh-N, which lacks the cholesterol modification. In polarized epithelial cells ectopic *hmgcr* would be expected to promote the basolateral release of Hh-Np and enhance its long distance transmission [13], [21], [22]. By contrast, ectopic *hmgcr* should have less of an effect on the release or transmission of the partially modified Hh protein Hh-N, which is thought to signal most effectively apically. While it is not clear how the subcellular organization of the secretion machinery in neurons might be related to that in polarized epithelial cells, the *hmgcr*-dependent PGC attractant must nevertheless be released from neuronal cells in a manner that enables it to move readily into and then through the underlying layer of mesodermal cells so that it can contact the migrating PGCs. If a key function of *hmgcr* in generating the ectopic attractant in neurons is to enhance the release and transmission of Hh into the underlying mesodermal layer then the same distinction should hold in PGC migration. In this case, co-expression of wild type Hh-Np would be expected to exacerbate the *hmgcr* induced migration defects, while co-expression of Hh-N should have more modest effects. On the other hand, if the germ cell attractant generated by ectopic *hmgcr* is completely independent of *hh*, then co-expression of either Hh ligand should contribute nothing to the *hmgcr* induced PGC migration defects.

To test these predictions we first compared the effects of driving *hh* or *hh-N* expression in the nervous system on PGC migration. Figure S2 shows that expression of a *UAS-hh* transgene in the nervous system using an *elav-GAL4* driver has a greater effect on PGC migration than a *UAS-hh-N* (3^nd^ chromosome) transgene encoding the truncated and partially modified Hh-N protein. We next examined the effects of co-expressing Hmgcr with either Hh-Np or Hh-N. Figure 7 shows that co-expression of Hh-N (3^rd^) and Hmgcr leads to only a small increase in the number of mismigrated PGCs. To confirm this finding, we tested a second *UAS-hh-N* transgene (2^nd^ chromosome). It gave comparable results. In contrast to the *UAS-hh-N* transgenes, a transgene expressing wild type Hh-Np collaborates with *hmgcr*, substantially augmenting the effects of ectopic *hmgcr* on PGC migration. In fact, the effects of the Hh-Np transgene are just about equivalent to that seen with two copies of *hmgcr*.

### *hmgcr* potentiates the release/transmission of *hh*-GFP

A strong prediction of the findings described above is that ectopic expression of Hmgcr in neuronal cells will enhance the release and/or transmission of the wild type Hh ligand from these cells into and through the underlying mesodermal cell layer. While we have previously visualized the effects of ectopic Hmgcr on Hh-Np release/transmission in the ectoderm using Hh antibodies [21], it was impossible to obtain reliable images of its transmission through the underlying mesoderm using these antibodies. For this reason, we turned to Hh-GFP to test this prediction. As described above, we found that Hh-GFP is less effective than either Hh-Np or Hh-N in disrupting PGC migration when expressed using the *twi-GAL4* driver. A similar result is obtained when the *elav-GAL4* driver was used to direct Hh-GFP expression in the embryonic nervous system (Figure S2). On the other hand, Figure 7 shows that Hh-GFP can potentiate the effects of Hmgcr when the two proteins are co-expressed in the nervous system. Thus, though Hh-GFP clearly doesn't fully recapitulate the activity of Hh-Np in this assay, it does resemble Hh-Np in being able to collaborate with Hmgcr.

**Figure 8:**
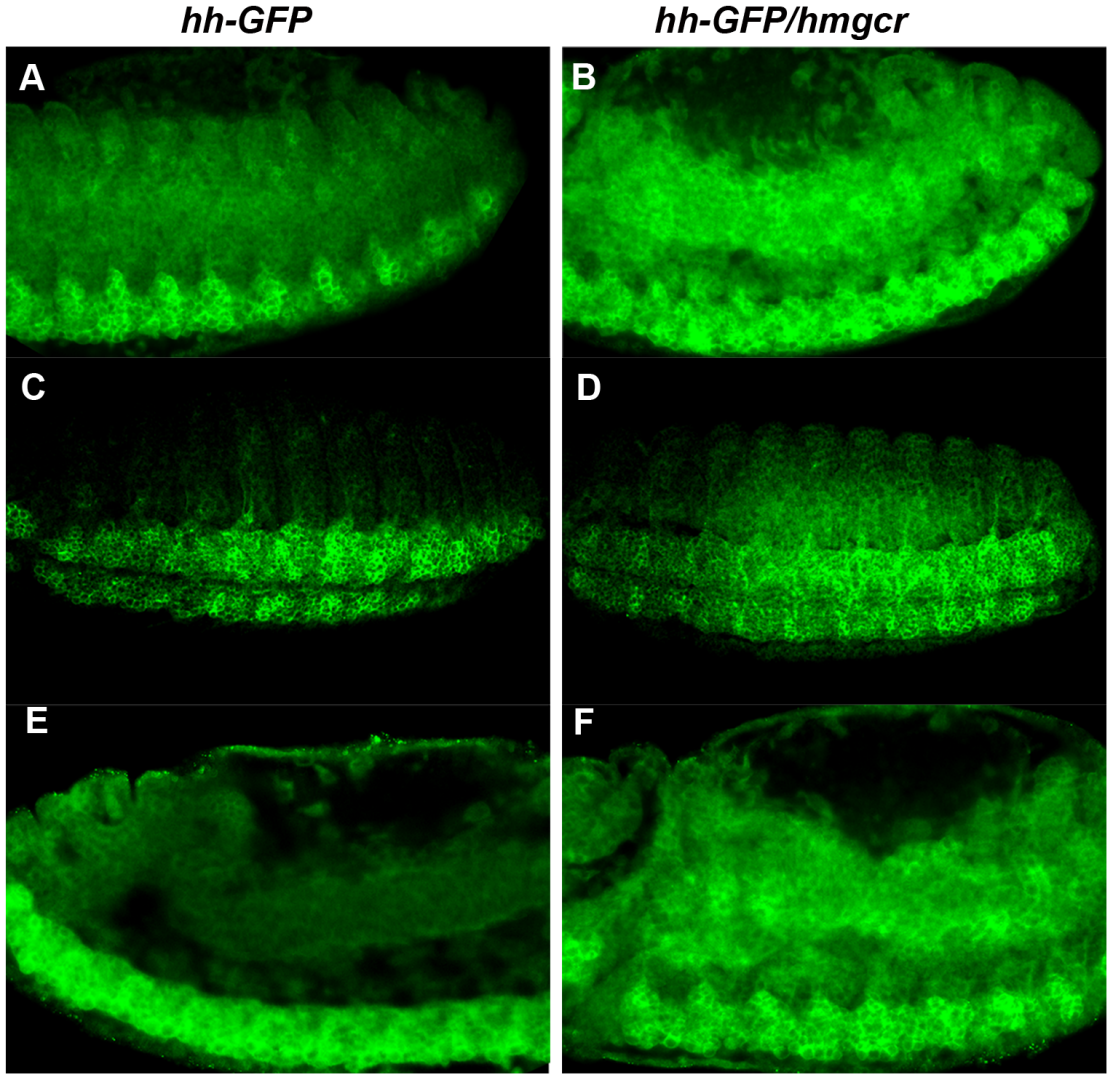
Co-expression of *hmgcr* with *hh* potentiates the transmission of the Hh ligand into and through the mesoderm. Homozygous *UAS-Hh-GFP* males were mated to females carrying an *elav-GAL4* transgene with or without *UAS-hmgcr* and the progeny probed with GFP antibody. Shown in panels A,C and E are *elav-GAL4/UAS-hh-GFP* embryos. In this experiment *elav-GAL4/CyO,actin-GFP* females were mated to homozygous *UAS-Hh-GFP* males. Embryos of the correct genotype were identified by the unique patterns of GFP expression generated by *actin-GFP* or the *elav/Hh-GFP* combination. Shown in panels B, D and F are *hh-GFP/hmgcr* embryos. Females homozygous for the recombinant *elav-GAL4*, *UAS-hmgcr* chromosome were mated to males homozygous for *UAS-Hh-GFP*. Panels A and B: early stage 13 embryos. Panels C and D: stage 14 embryos. Panels E and F: late stage 14 embryos.

To determine whether Hmgcr promotes the transmission of Hh into the underlying tissue, we compared the Hh-GFP distribution in embryos expressing this fusion protein in the nervous system with or without Hmgcr. We first detect Hh-GFP in the developing PNS in stage 9–10 embryos, while it subsequently comes on in the developing CNS in stage 11–12 embryos. At these early stages the level of Hh-GFP is relatively low, and the differences in Hh-GFP distribution between *hh-GFP* and *hh-GFP/hmgcr* embryos are not very pronounced. This is likely due in part to residual Hmgcr activity from earlier zygotic expression and from maternal deposition and in part because only small amounts of ectopic Hmgcr and Hh-GFP have been synthesized. However, as development proceeds (and Hh-GFP levels increase) the differences between *hh-GFP* and *hh-GFP/hmgcr* embryos become more pronounced. This is illustrated in Figure 8. Panels A and B show cross sections of stage 13 *hh-GFP* and *hh-GFP/hmgcr* embryos; panels C and D show views of the surface of stage 14 embryos; while panels E and F show cross sections of stage 14 embryos. The same embryos, together with a series of plots of the average pixel density across several ∼3 cell wide sections (as indicated), are shown in Figures S3–S5.

Two differences between *hh-GFP* and *hh-GFP/hmgcr* embryos are apparent. First, the overall level of Hh-GFP is elevated in the *hh-GFP/hmgcr* embryos. In scans of several similarly staged and oriented embryos there was a ∼1.8–2.0 fold difference in the Hh-GFP signal between *hh-GFP* and *hh-GFP/hmgcr*. Since different *elav-GAL4* transgenes (one on the 2^nd^ and the other recombined with *UAS-hmgcr* on the 3^rd^) were used to drive *UAS-hh-GFP* expression, one explanation for this difference is that the recombined *elav-GAL4* transgene drives a higher level of expression. To test this we used the two *elav-GAL4* transgenes to express GFP carrying a nuclear localization signal. As shown in Figure S6, the two drivers produce similar amounts of GFP. Thus, a more likely possibility is that *hmgcr* stabilizes or otherwise promotes Hh accumulation. Consistent with this explanation, we found that the level of endogenous Hh-Np in the ectoderm increased to a similar if not greater extent when *hmgcr* was ectopically expressed using a *hh-GAL4* driver [19]. Second, the transmission of Hh-GFP from expressing cells in the nervous system into the surrounding ectoderm and through the underlying mesoderm is substantially enhanced by co-expression of Hmgcr. This is most obvious in the two sets of stage 14 embryos shown in panels C–F (see also Figures S4 and S5). In both cases, Hh-GFP is largely restricted to the CNS in *hh-GFP* embryos. In contrast, Hh-GFP is found not only in the nervous system but also in the adjacent ectoderm (Figure 8C and D) and, most importantly, in the underlying mesodermal cell layer in the *hh-GFP/hmgcr* embryos (Figure 8E and F). Although less pronounced, differences in the level and distribution of Hh-GFP are also evident in stage 13 embryos. This can be seen by comparing panels A and E versus B and F in Figure 8 and the plots of pixel density in Figure S3 and S5.

**Figure 9:**
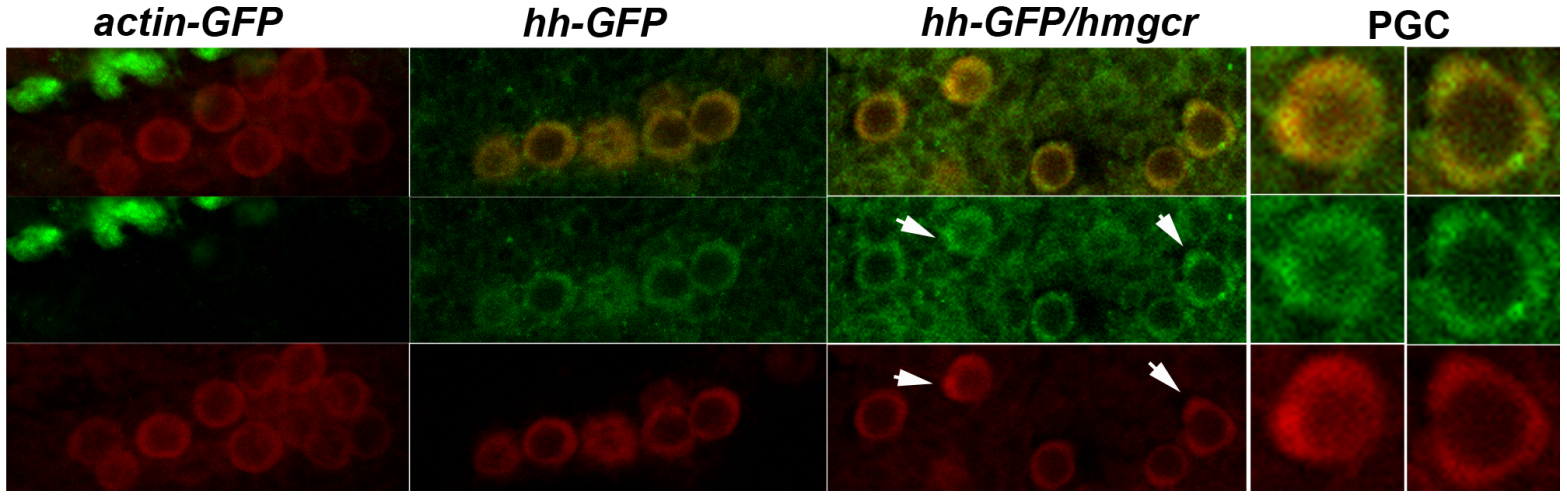
Ectopically expressed Hh-GFP localizes near PGCs. *elav-GAL4/CyO,actin-GFP* females were mated to homozygous *UAS-Hh-GFP* males. Embryos carrying the *CyO*, *actin-GFP* balancer and *UAS-Hh-GFP* but no GAL4 driver were identified by their distinctive GFP expression pattern, and the PGCs from one of these embryos are shown in the first set of panels as indicated. The second set of panels show PGCs from the sibling *elav-GAL4/UAS-Hh-GFP* embryos. In the third set of panels females homozygous for the recombinant *elav-Gal4*, *UAS-hmgcr* chromosome were mated to males homozygous for *UAS-Hh-GFP*. Embryos of the indicated genotypes (*actin-GFP*: *CyO,Actin-GFP/UAS-Hh-GFP*; *hh-GFP*: *elav-GAL4/UAS-hh-GFP*; *hh-GFP/hmgcr*: *elav-Gal4*, *UAS-hmgcr/UAS-hh-GFP*) were stained with GFP (imaged in green) and Vasa (imaged in red) antibodies. Top panels show merged images, middle panels Hh-GFP, and bottom panels Vasa. The PGCs shown in all three sets are from stage 13 embryos. Arrows in the *hh-GFP/hmgcr* panels indicate PGCs with bulges/protrusions enriched for Hh-GFP. Panels on far right show close-ups of the two PGCs in the *hh-GFP/hmgcr* panels that are marked with arrows.

In addition to being transmitted from the developing nervous system into the mesoderm, we found that Hh-GFP localizes preferentially to PGCs or to tissues containing PGCs. Figure 9 shows PGCs in stage 12–13 embryos expressing Actin-GFP, Hh-GFP or Hh-GFP/Hmgcr. During this period of development PGCs in wild type embryos associate and then align with the gonadal mesodermal SGPs. In both the *actin-GFP* and *hh-GFP* embryos, the SGPs are aligned with the gonadal mesoderm, while in the *hh-GFP/hmgcr* embryos they are scattered in the mesoderm. The middle set of panels shows that PGCs in both the *hh-GFP* and *hh-GFP/hmgcr* are distinctly outlined with Hh-GFP, while a GFP signal is not associated with PGCs in the control *actin-GFP*/*UAS-hh-GFP* embryos. The PGC associated GFP signal appears earlier in *hh-GFP/hmgcr* embryos and is (together with the signal in the surrounding mesodermal tissue: see Figure 9) greater than in *hh-GFP* embryos. In addition, we often observed PGCs in the *hh-GFP/hmgcr* embryos that have bulges or protrusions. As indicated by the arrows in the *hh-GFP/hmgcr* panels and in the adjacent enlargements of these PGCs, there are higher levels of Hh-GFP associated with these bulges. However, not all concentrations of Hh-GFP are found associated with obvious bulges or protrusions.

## Discussion

The synthesis of mevalonic acid by the enzyme Hmgcr is the rate-controlling step in the biosynthesis of isoprenoids and steroids. In mammals, one end-product of the mevalonate pathway, cholesterol, is used to modify the C-terminus of the processed Hh ligand, and this modification plays an important role in controlling the activity of this signaling molecule. Flies lack the enzymes needed for *de novo* cholesterol biosynthesis and depend instead upon exogenous cholesterol for this modification of the Hh ligand [24]. Nevertheless, the mevalonate biosynthetic pathway is still used to potentiate the release/transmission of the Hh ligand, in this case through (at least in part) the geranylation of the G protein Gγ1 [21], [22], [35]. Hmgcr as well as the downstream components in the isoprenoid biosynthetic pathway also play a pivotal role in generating the attractant that guides PGC migration both from its native source, the SGPs, and from a variety of different embryonic tissues when ectopically expressed [27]–[30]. However, how *hmgcr* or the other isoprenoid pathway enzymes function in generating the PGC attractant either in the SGPs or at ectopic sites has remained unresolved and contentious. To address this problem we have focused on the connection between the mevalonate→isoprenoid biosynthetic pathway and two proteins that have been implicated in the long distance basolateral transmission of the Hh-Np ligand, the G protein Gγ1 and the extracellular *hh* signaling factor Shf.

Previous studies have established that a rate limiting step in generating the PGC attractant either by the SGPs or by other tissues and cell types is the biosynthesis of geranylgeranyl-pyrophosphate by geranylgeranyl diphosphate synthetase (*qm*) [24], [27], [28]. The control point in the geranylgeranyl-pyrophosphate biosynthetic pathway is the production of mevalonic acid by the enzyme Hmgcr. While *hmgcr* seems to play a rather similar role in the release/transmission of Hh-Np from *hh* sending cells, in this case through the geranylation of Gγ1, an important and controversial question is whether the functioning of the *hmgcr→qm* biosynthetic pathway in *hh* signaling has any connection to the generation of the PGC attractant. We have addressed this question by determining if the PGC migration defects induced by *hmgcr* expression in the nervous systems depend upon Gγ1 and Shf. We find that mutations in both *gγ1* and *shf* dominantly suppress the migration defects induced by ectopic *hmgcr*. In contrast, reducing the dose of the *hmgcr* gene dominantly enhances the migration defects induced by *hmgcr* expression in the nervous system. This later finding is expected since reducing *hmgcr* activity in the SGPs should further compromise the ability of the attractant generated by the SGPs to compete with the attractant generated in the nervous system. The former findings show that the production/activity of the attractant generated in the nervous system by ectopic *hmgcr* depends on both *gγ1* and *shf*. By themselves, these results do not exclude the possibility that *gγ1* and *shf* only collaborate with *hmgcr* when it is ectopically expressed in the nervous system while they are not actually needed for the *hmgcr*-dependent production of the attractant by the SGPs. However, this scenario seems unlikely. For one, there are PGC migration defects in *gγ1* and *shf* mutant embryos. For another, the Gγ1 protein must be geranylated to function in PGC migration [22]. Finally, like *hmgcr*, ectopic expression of *gγ1* and *shf* in the mesoderm and ectoderm perturbs PGC migration.

Even though Gγ1 and Shf are known to function in the release and transmission of the Hh ligand, it could be argued that these two proteins could also mediate the release/transmission of others molecules, including the “actual” PGC attractant. Indeed, Gγ1 is likely involved in secretion of other molecules, while the fact that Shf homologs in mammals function in Wnt but not Hh signaling raises the possibility that Shf could promote signaling by an as yet unknown ligand (though not Wg: [34], [40]). However, there is evidence that like Gγ1 and Shf, Hh itself depends upon *hmgcr* and the isoprenoid biosynthetic pathway not only in *hh* signaling but also in generating an ectopic PGC attractant in the nervous system. This comes from the differences in the effects of ectopically expressed Hh-Np and Hh-N that would be predicted based on the mechanisms proposed for their transmission [15], [20]. First, the apically transmitted Hh-N ligand would be expected to have a smaller effect on PGC migration when ectopically expressed in the nervous system than Hh-Np. With the caveat that expression of different *UAS* transgene inserts will not be precisely the same, this prediction holds. Second, the geranylation of Gγ1 in response to ectopic Hmgcr would be expected to promote the basolateral release and subsequent spreading of Hh-Np into the mesoderm. By contrast, ectopic Hmgcr should have less influence on Hh-N, which isn't readily internalized by *hh* sending cells and spreads mostly along the apical surface [20]. With the same caveat, this predicted distinction is also observed. When co-expressed, Hh-Np and Hmgcr collaborate to strongly potentiate PGC migration defects, while there is a more modest collaboration between Hh-N and Hmgcr.

Though an imperfect mimic of Hh-Np, we have taken advantage of a chimeric Hh-GFP fusion protein to analyze the effects of Hmgcr on the transmission of Hh from cells in the embryonic nervous system. We found that Hh-GFP is less effective than Hh-Np (and even Hh-N) in competing with the PGC attractant produced by the SGPs when it is ectopically expressed using the *twi* or *elav GAL4* drivers. Since Hh-GFP appears to have near but not quite wild type activity in morphogenesis [39], it is surprising that it is relatively ineffective in altering PGC migration. However, a plausible reason for this discrepancy is that the demands imposed by the assays used to test Hh-GFP activity in each experimental context are quite different. The morphogenesis assay requires that Hh-GFP substitute for Hh-Np. Since animals can readily tolerate heterozygosity for *hh*, small deficits in the functioning of the chimeric protein might only have minimal effects on morphogenesis. In contrast, in the PGC migration assay the ectopically expressed Hh-GFP must be able to compete with the attractant(s) produced by the SGPs. If Hh-Np is the relevant endogenous PGC attractant, then even subtle deficiencies in the activity of the chimeric Hh-GFP ligand would be expected to compromise its ability to compete with the wild type protein. It would also follow that it should be possible to “rescue” ectopic Hh-GFP by enhancing its activity. This is the case. While *hh-GFP* is not very active on its own, it is able to collaborate with *hmgcr* when co-expressed in the nervous system.

In previous studies we have shown that expressing *hmgcr* in *hh* producing cells in the ectoderm increases the overall level of Hh protein and enhances its transmission to adjacent cells [21]. Precisely the same sorts of effects on Hh-GFP are evident when it is “rescued” by co-expression with *hmgcr* in the nervous system—Hh-GFP levels are elevated, while its transmission into and through the underlying mesodermal cell layer is appreciably enhanced. These *hmgcr* dependent effects, particularly on the movement of Hh-GFP from the neuroectoderm into the underlying mesoderm, would also provide a plausible explanation for why this biosynthetic enzyme plays such a pivotal role in PGC migration even though it is not directly responsible for the synthesis of the PGC attractant. In the period when PGCs are migrating through the mesoderm, the SPGs are the only cells in the embryo simultaneously expressing both *hmgcr* and *hh* [27], [28], [31]. Consequently the accumulation, release and transmission of Hh-Np will be specifically potentiated in SGPs, but not in other *hh* expressing cells elsewhere in the mesoderm or in the ectoderm. This would provide a mechanism for ensuring that SGP derived Hh-Np out-competes Hh-Np produced elsewhere. Taken together, these findings support the idea that Hh-Np expressed in the SGPs functions as a PGC attractant. With the caveat that the activities of Hh-GFP are not identical to Hh-Np, the fact that Hh-GFP accumulates on the surface and around the PGCs further bolsters this suggestion. Moreover, in a subset of the PGCs Hh-GFP is closely associated with bulges or protrusions that could potentially be of relevance to the process of migration.

On the other hand, a number of critical questions remain. For one, it is not clear how reception of the *hh* signal could actually translate into directed movement. The endpoint of the signaling cascade in the canonical pathway is the transcriptional activation of target genes, including the *hh* receptor *ptc*. However, transcription is likely not involved in this instance, as *ptc* reporters are not activated in PGCs [29] (unpublished data). Moreover, in mammals *hh* dependent axonal guidance and fibroblast migration are independent of transcription and involve instead the coupling of Smo activation to pathways that mediate the reorganization of the cytoskeleton [6], [7], [41], [42]. Further studies will clearly be required to establish a connection between *hh* signaling to the PGCs, changes in the cytoskeleton and directed movement. Another unresolved question is whether SGPs produce any other PGC attractants. Although no other plausible candidates have been identified, our experiments do not exclude the possibility that there are other PGC attractants, even including an attractant(s) whose activity, like Hh-Np, is potentiated by the *hmgcr* isoprenoid biosynthetic pathway.

## Materials and Methods

### Immunohistochemistry

The embryo stainings were performed essentially as described in 21. Vasa (from Paul Lasko) and Hh (from Tom Kornberg) antibodies are rabbit polyclonal antibodies. Both were used at a 1∶500 dilution. Engrailed and Wingless antibodies are mouse monoclonal antibodies and were used at 1∶10 dilution. ß-Galactosidase antibody was either a rabbit polyclonal purchased from Cappel (used at 1∶1000 dilution) or a mouse monoclonal antibody from Developmental Studies Hybridoma Bank (used at 1∶10 dilution). GFP antibody is a rabbit polyclonal purchased from Torrey Pines Biolabs (used at 1∶1000 dilution). For con-focal analysis a magnification of 40× was used in almost all the instances, and images were collected using identical settings for the control and experimental samples. Multiple pairs of wild type (sibs) and mutant embryos were imaged in each case, and representative examples are presented.

### Mutant and misexpression analysis

*Shf* stocks including mutants and *UAS* transgenes were obtained from Seth Blair [32]. *UAS-Hh-GFP* fusion stocks were a kind gift of Isabel Guerrero [20]. *UAS-Hh-Np* (two different stocks on the second and third chromosome respectively; from Phil Beachy), *UAS-Hh-N*: two different stocks on the second (from Tom Kornberg) and third chromosome (from Phil Beachy) respectively, *gγ1* mutant stocks, *gγ1^N159^* and *gγ1* ^k0817^, were obtained from Fumio Matsuzaki, while *UAS*- *gγ1* stock (*gγ1*) was kindly provided by the Olson lab [43]. The other *UAS* and *Gal4* stocks used for the misexpression studies were from the Bloomington stock center. *hairy-Gal4*, *elav-Gal4*, *nanos*-*Gal4*, *patched-Gal4*, *UAS-ß-galactosidase*, *hh-Gal4*/*TM6 Ubx-LacZ*. In most experiments, males carrying two of the copies *UAS* transgene were mated with virgin females carrying two copies of the *Gal4* transgene. The resulting progeny embryos were fixed and stained for subsequent analysis. *elav-Gal4*, *UAS hmgcr* recombinant [38] and *UAS-qm* stock [24] were obtained from Ruth Lehmann. In many experiments the genotypes of the embryos were unambiguously determined by using balancer chromosomes marked with either GFP or ß-galactosidase.

## Supporting Information

Figure S1 Overexpression of *shf* elevates level of Wingless (Wg) accumulation. Females homozygous for the *UAS-shf* transgene were mated with *hh-GAL4*/TM6 *Ubx-LacZ* males. Embryos generated from the cross were collected and coimmunostained with Wg (imaged in red) and β-galactosidase (imaged in green, not shown) antibodies. (A) control *UAS-shf/Ubx-LacZ*. (B) *hh-GAL4/UAS-shf*.
(TIF)

Figure S2 Effects of ectopic expression of different Hh proteins in the nervous system. An *elav-GAL4* driver inserted on the 2^nd^ chromosome (as described in the text and the legend to Figure 7) was used to drive expression of *UAS-hh*, *UAS-hh-N* (the *UAS-hh-N* transgenic line on the 3^rd^ chromosome in Figure 7), and *UAS-hh-GFP*. The graph shows the % of embryos having different numbers of mismigrated PGCs as indicated in the Figure.
(TIF)

Figure S3 Distribution of Hh-GFP in stage 13 hh*-GFP* and *hh-GFP/hmgcr* embryos. Embryos in Figure S3 are from panels A and B of Figure 8. This supplemental figure shows a plot of the pixel density (using ImageJ) in an approximately 3 cell wide vertical stripe. The center of each of these stripes is indicated by the arrows below each embryo. There are four plots for each embryo.
(TIF)

Figure S4 Distribution of Hh-GFP in stage14 *hh-GFP* and *hh-GFP/hmgcr* embryos. Embryos in Figure S4 are from panels C and D of Figure 8. This supplemental figure shows a plot of the pixel density (using ImageJ) in an approximately 3 cell wide vertical stripe. The center of each of these stripes is indicated by the arrows below each embryo. There are four plots for each embryo.
(TIF)

Figure S5 Distribution of Hh-GFP in stage 14 *hh-GFP* and *hh-GFP/hmgcr* embryos. Embryos in Figure S5 are from panels E and F of Figure 8. This supplemental figure has a plot of the pixel density (using ImageJ) in an approximately 3 cell wide vertical stripe. The center of each of these stripes is indicated by the arrows below each embryo. There are four plots for each embryo.
(TIF)

Figure S6 The 2^nd^ and recombined 3^rd^ chromosome *elav-GAL4* transgenes drive equivalent levels of expression of a *UAS-GFP(NLS)* reporter. Females homozygous for the recombined *UAS-hmgcr*, *elav-GAL4* 3^rd^ chromosome or the 2^nd^ chromosome *elav-GAL4* driver (*elav-GAL4*/*CyO Actin GFP*) were mated to *UAS-GFP(NLS)* males. The resulting embryos were fixed and stained with anti-GFP antibody. (A) *elav-GAL4* (2^nd^)/*UAS-GFP(NLS)*. (B) *UAS-hmgcr,elav-GAL4* (3^rd^)/*UAS-GFP(NLS)*. The signal intensity in each nuclei was estimated by doing pixel counts over a fixed area using ImageJ software. The average pixel intensity per nucleus for *elav-Gal4*/*UAS-GFP(NLS)* was 69.8 (S.D.+/−9.7; n = 15) whereas for *UAS-hmgcr,elavGal4* embryos was 66 (S.D.+/−13.5; n = 15).
(TIF)

Text S1 Does ectopic Hh perturb germ cell migration? The data in Figures 5, 7 and S2 showing that *hh* misexpression perturbs PGC migration are contradicted by experiments reported in Figure 2 of a paper “Hedgehog does not guide migrating Drosophila germ cells” that was published in 2009 by Renault *et al.* [29] in *Developmental Biology*. The material presented in Text S1 addresses this controversy.
(PDF)

## References

[1] Huangfu D, Anderson KV (2006) Signaling from Smo to Ci/Gli: conservation and divergence of Hedgehog pathways from *Drosophila* to vertebrates. Development 33: 3–14. 16339192 10.1242/dev.02169

[2] Matise M (2007) Order in the classroom: graded responses to instructive Hh signaling in the CNS. Cell Cycle 10: 1194–9. 17495537 10.4161/cc.6.10.4249

[3] Wilson CW, Chuang PT (2010) Mechanism and evolution of cytosolic Hedgehog signal transduction. Development 137: 2079–94. 20530542 10.1242/dev.045021 PMC2882129

[4] Matise MP, Wang H (2011) Sonic hedgehog signaling in the developing CNS where it has been and where it is going. Curr Top Dev Biol 97: 75–117. 22074603 10.1016/B978-0-12-385975-4.00010-3

[5] Testaz S, Jarov A, Williams KP, Ling LE, Koteliansky VE, et al (2001) Sonic hedgehog restricts adhesion and migration of neural crest cells independently of the Patched-Smoothen-Gil signaling pathway. Proc Natl Acad Sci U S A 98: 12521–12526. 11592978 10.1073/pnas.221108698 PMC60086

[6] Hochman E, Castiel A, Jacob-Hirsch J, Amariglio N, Izraeli S (2006) Molecular pathways regulating pro-migratory effects of Hedgehog signaling. J Biol Chem 281 33860–70. 16943197 10.1074/jbc.M605905200

[7] Charron F, Stein E, Jeong J, McMahon AP, Tessier-Lavigne M (2003) The morphogen sonic hedgehog is an axonal chemoattractant that collaborates with netrin-1 in midline axon guidance. Cell 113: 11–23. 12679031 10.1016/s0092-8674(03)00199-5

[8] Deshpande G, Swanhart L, Chiang P, Schedl P (2001) Hedgehog signaling in germ cell migration. Cell 106: 759–69. 11572781 10.1016/s0092-8674(01)00488-3

[9] Kato K, Chihara T, Hayashi S (2004) Hedgehog and decapentabplegic instruct polarized growth of cell extensions in the *Drosophila* trachea. Development (Camb.) 131: 5253–5261. 10.1242/dev.01404 15456724

[10] Mann RK, Beach PA (2004) Novel lipid modifications of secreted protein signals. Annu Rev Biochem 2004 73: 891–923. 10.1146/annurev.biochem.73.011303.073933 15189162

[11] Gallet A (2011) Hedgehog morphogen: from secretion to reception. Trends Cell Biol 21: 238–246. 21257310 10.1016/j.tcb.2010.12.005

[12] Gallet A, Rodriguez R, Ruel L, Therond PP (2003) Cholesterol modification of hedgehog is required for trafficking and movement, revealing an asymmetric cellular response to hedgehog. Dev Cell 4: 191–204. 12586063 10.1016/s1534-5807(03)00031-5

[13] Avanesov A, Blair SS (2013) The *Drosophila* WIF1 homolog Shifted maintainsglypican-independent Hedgehog signaling and interacts with the Hedgehog co-receptors Ihog and Boi. Development 140: 107–16. 23154411 10.1242/dev.078444 PMC3513995

[14] Bilioni A, Sanchez-Hernandez D, Callejo A, Gradilla AC, Ibanez C, et al (2013) Balancing Hedgehog, a retention and release equilibrium given by Dally, Ihog, Boi and shifted/DmWif. Dev Biol 376: 198–221. 23276604 10.1016/j.ydbio.2012.12.013

[15] Callejo A, Torroja C, Quijada L, Guerrero I (2006) Hedgehog lipid modifications are required for Hedgehog stabilization in the extracellular matrix. Development 133: 471–83. 16396909 10.1242/dev.02217

[16] Gallet A, Ruel L, Staccini-Lavenant L, Thérond PP (2006) Cholesterol modification is necessary for controlled planar long-range activity of Hedgehog in *Drosophila* epithelia. Development1 33: 407–18. 10.1242/dev.02212 16396912

[17] The I, Bellaiche Y, Perrimon N (1999) Hedgehog movement is regulated through tout velu-dependent synthesis of a heparan sulfate proteoglycan. Mol Cell 4: 633–9. 10549295 10.1016/s1097-2765(00)80214-2

[18] Häcker U, Nybakken K, Perrimon N (2006) Heparan sulphate proteoglycans: the sweet side of development. Nat Rev Mol Cell Biol 6: 530–41. 16072037 10.1038/nrm1681

[19] Burke R, Nellen D, Bellotto M, Hafen E, Senti KA, et al (1999) Dispatched, a novel sterol-sensing domain protein dedicated to the release of cholesterol-modified hedgehog from signaling cells. Cell 99: 803–15. 10619433 10.1016/s0092-8674(00)81677-3

[20] Callejo A, Bilioni A, Mollica E, Gorfinkiel N, Andrés G, et al (2011) Dispatched mediates Hedgehog basolateral release to form the long-range morphogenetic gradient in the *Drosophila* wing disk epithelium. Proc Natl Acad Sci U S A 108: 12591–8. 21690386 10.1073/pnas.1106881108 PMC3150953

[21] Deshpande G, Schedl P (2005) HMGCoA reductase potentiates hedgehog signaling in *Drosophila melanogaster* . Dev Cell 9: 629–38. 16256738 10.1016/j.devcel.2005.09.014

[22] Deshpande G, Godishala A, Schedl P (2009) Gγ1, a downstream target for the *hmgcr*-isoprenoid biosynthetic pathway, is required for releasing the Hedgehog ligand and directing germ cell migration. Plos Genetics 5 (1) e1000333 doi:10.1371/journal.pgen.1000333 19132091 10.1371/journal.pgen.1000333 PMC2607556

[23] DeBose-Boyd RA (2008) Feedback regulation of cholesterol synthesis: sterol-accelerated ubiquitination and degradation of HMG CoA reductase. Cell Res 18: 609–621. 18504457 10.1038/cr.2008.61 PMC2742364

[24] Santos AC, Lehmann R (2004) Isoprenoids control germ cell migration downstream of HMGCoA reductase. Dev Cell 6: 283–93. 14960281 10.1016/s1534-5807(04)00023-1

[25] Anel AMD, Malhotra V (2005) PKCn is required for b1g2/b3g2 and PKD-mediated transport to the cell surface and the organization of the Golgi apparatus. Jour Cell Biol 169: 83–91. 15824133 10.1083/jcb.200412089 PMC2171908

[26] Bard F, Malhotra V (2006) The formation of TGN to plasma membrane transport carriers. Annu Rev Cell Dev Biol 232: 439–55. 16824007 10.1146/annurev.cellbio.21.012704.133126

[27] Van Doren M, Broihier HT, Moore LA, Lehmann R (1998) HMG-CoA reductase guides migrating primordial germ cells. Nature 396: 466–9. 9853754 10.1038/24871

[28] Kunwar PS, Siekhaus DE, Lehmann R (2006) In vivo migration: A germ cell perspective. Annual Review of Cell. And Dev Biol 22: 237–65. 10.1146/annurev.cellbio.22.010305.103337 16774460

[29] Renault AD, Ricardo S, Kunwar PS, Santos A, Starz-Gaiano M, et al (2009) Hedgehog does not guide migrating *Drosophila* germ cells. Dev Biol 328: 355–62. 19389345 10.1016/j.ydbio.2009.01.042 PMC2693393

[30] Richardson BE, Lehmann R (2010) Mechanisms guiding primordial germ cell migration: strategies from different organisms. Nat Rev Mol Cell Biol 11: 37–49. 20027186 10.1038/nrm2815 PMC4521894

[31] Hsieh JC, Kodjabachian L, Rebbert ML, Rattner A, Smallwood PM, et al (1999) A new secreted protein that binds to Wnt proteins and inhibits their activities. Nature 398: 431–6. 10201374 10.1038/18899

[32] Glise B, Miller CA, Crozatier M, Halbisen MA, Wise S, et al (2005) Shifted, the *Drosophila* ortholog of Wnt inhibitory factor-1, controls the distribution and movement of Hedgehog. Dev Cell 8: 255–66. 15691766 10.1016/j.devcel.2005.01.003

[33] Gorfinkiel N, Sierra J, Callejo A, Ibañez C, Guerrero I (2005) The *Drosophila* ortholog of the human Wnt inhibitor factor Shifted controls the diffusion of lipid-modified Hedgehog. Dev Cell 8: 241–53. 15691765 10.1016/j.devcel.2004.12.018

[34] Avanesov A, Honeyager SM, Malicki J, Blair SS (2012) The role of glypicans in Wnt inhibitory factor-1 activity and the structural basis of Wif1's effects on Wnt and Hedgehog signaling. PLoS Genet 8 (2) e1002503. 22383891 10.1371/journal.pgen.1002503 PMC3285576

[35] Callejo A, Culi J, Guerrero I (2007). Lipids and Lipoproteins as carriers for Hedgehog spreading and reception. Fly Meeting Abstracts. Drosophilia Research Conference. March 7–11, 2007. Philadelphia, Pennsylvania, United States of America.

[36] Delon I, Payre F (2004) Evolution of larval morphology in flies: get in shape with shavenbaby. Trends Genet 20: 305–13. 15219395 10.1016/j.tig.2004.05.003

[37] Zhai Z, Fuchs AL, Lohmann I (2010) Cellular analysis of newly identified Hox downstream genes in Drosophila. Eur J Cell Biol 89: 273–8. 20018403 10.1016/j.ejcb.2009.11.012

[38] Ricardo S, Lehmann R (2009) An ABC transporter controls export of a Drosophila germ cell attractant. Science 323: 943–946. 19213920 10.1126/science.1166239 PMC2729540

[39] Torroja C, Gorfinkiel N, Guerrero I (2004) Patched controls the Hedgehog gradient by endocytosis in a dynamin-dependent manner, but this internalization does not play a major role in signal transduction. Development 131: 2395–2408. 15102702 10.1242/dev.01102

[40] Sánchez-Hernández D, Sierra J, Ortigão-Farias JR, Guerrero I (2012) The WIF domain of the human and Drosophila Wif-1 secreted factors confers specificity for Wnt or Hedgehog. Development 2012 Sep 5. [Epub ahead of print]. 10.1242/dev.080028 22951645

[41] Bijlsma MF, Borensztajn KS, Roelink H, Peppelenbosch MP, Spek CA (2007) Sonic hedgehog induces transcription-independent cytoskeletal rearrangement and migration regulated by arachidonate metabolites. Cell Signal 19 2596–604. 17884337 10.1016/j.cellsig.2007.08.011

[42] Polizio AH, Chinchilla P, Chen X, Kim S, Manning DR, et al (2011) Heterotrimeric Gi proteins link Hedgehog signaling to activation of Rho small GTPases to promote fibroblast migration. J Biol Chem 286: 19589–96. 21474452 10.1074/jbc.M110.197111 PMC3103338

[43] Yi P, Han Z, Li X, Olson EN (2006) The mevalonate pathway controls heart formation in *Drosophila* by isoprenylation of Ggamma1. Science 313: 301–3. 16857902 10.1126/science.1127704

