# Supplementary figures and images for: The hedgehog Pathway Gene shifted Functions together with the hmgcr-Dependent Isoprenoid Biosynthetic Pathway to Orchestrate Germ Cell Migration
Source: PLoS Genet. 2013 Sep 12;9(9):e1003720. doi: 10.1371/journal.pgen.1003720 (PMC3772052; doi:10.1371/journal.pgen.1003720)

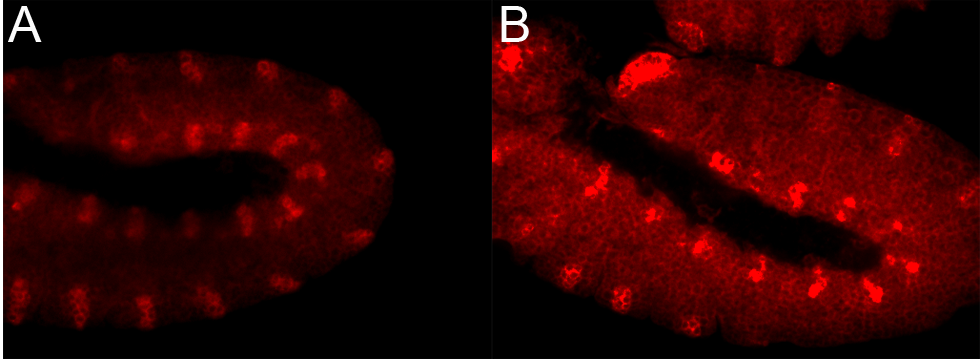

Supplement: Figure S1 — Overexpression of shf elevates level of Wingless (Wg) accumulation. Females homozygous for the UAS-shf transgene were mated with hh-GAL4/TM6 Ubx-LacZ males. Embryos generated from the cross were collected and coimmunostained with Wg (imaged in red) and β-galactosidase (imaged in green, not shown) antibodies. (A) control UAS-shf/Ubx-LacZ. (B) hh-GAL4/UAS-shf. (TIF) [file pgen.1003720.s001.tif]

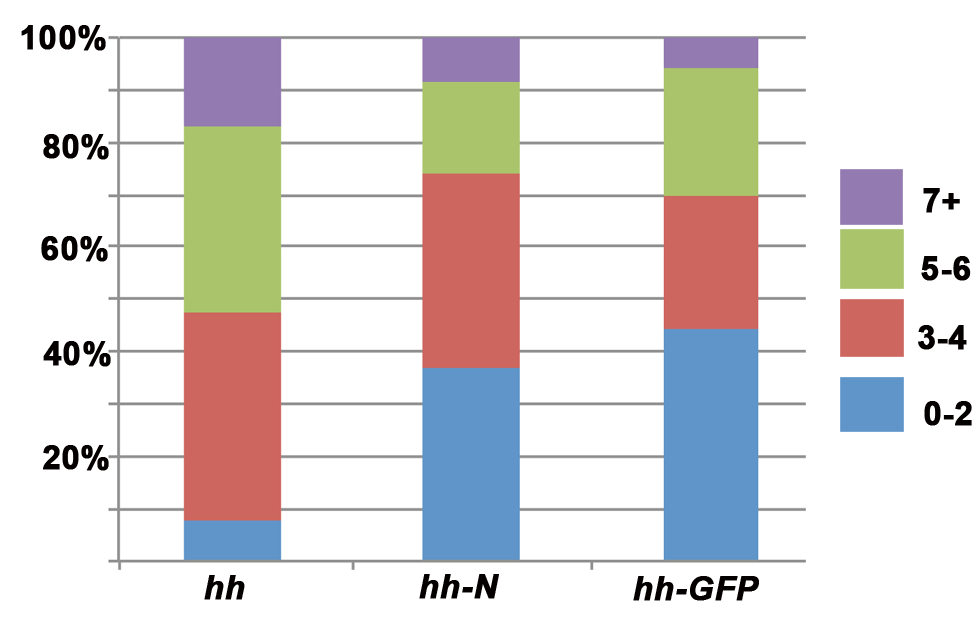

Supplement: Figure S2 — Effects of ectopic expression of different Hh proteins in the nervous system. An elav-GAL4 driver inserted on the 2nd chromosome (as described in the text and the legend to Figure 7) was used to drive expression of UAS-hh, UAS-hh-N (the UAS-hh-N transgenic line on the 3rd chromosome in Figure 7), and UAS-hh-GFP. The graph shows the % of embryos having different numbers of mismigrated PGCs as indicated in the Figure. (TIF) [file pgen.1003720.s002.tif]

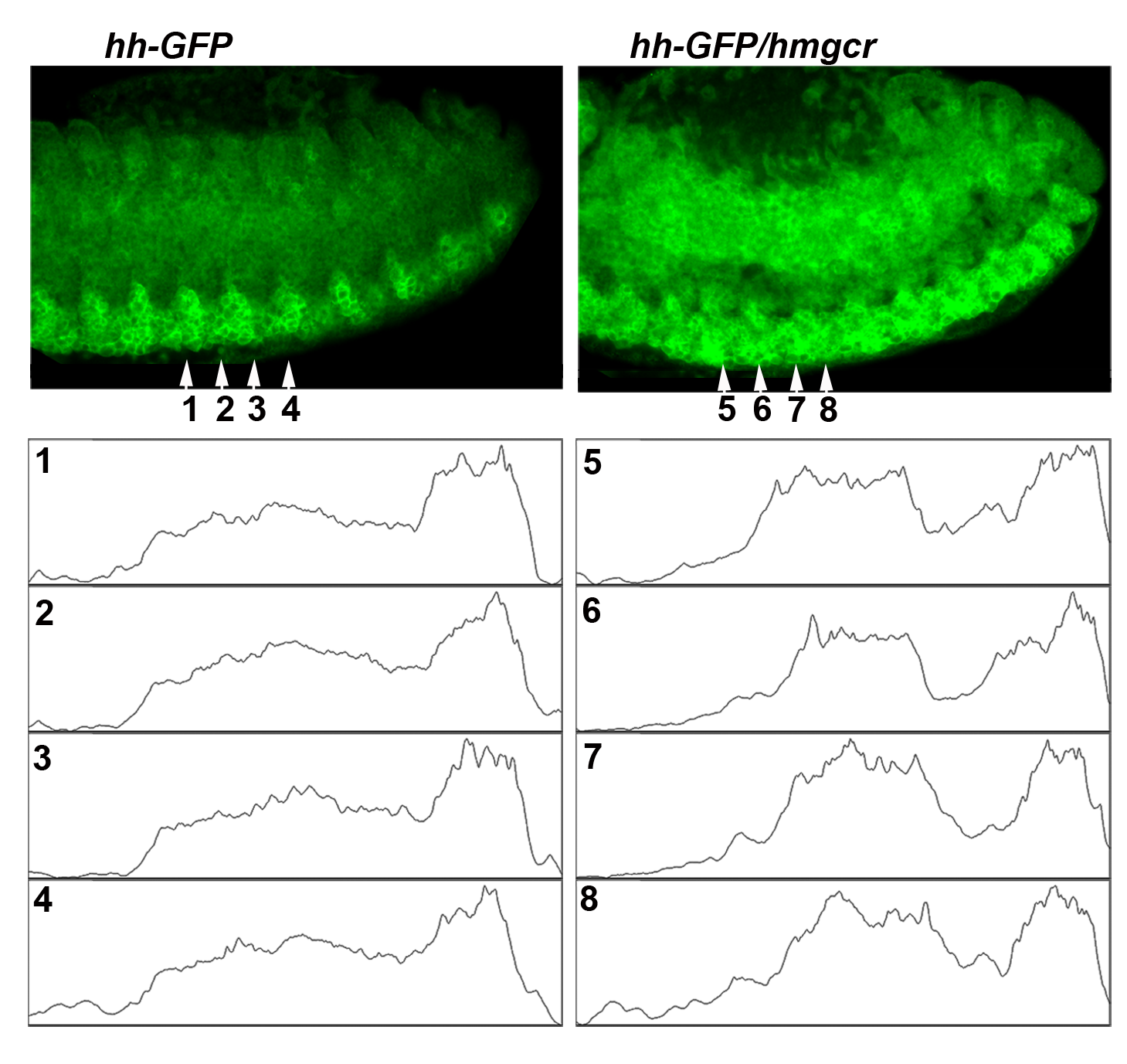

Supplement: Figure S3 — Distribution of Hh-GFP in stage 13 hh-GFP and hh-GFP/hmgcr embryos. Embryos in Figure S3 are from panels A and B of Figure 8. This supplemental figure shows a plot of the pixel density (using ImageJ) in an approximately 3 cell wide vertical stripe. The center of each of these stripes is indicated by the arrows below each embryo. There are four plots for each embryo. (TIF) [file pgen.1003720.s003.tif]

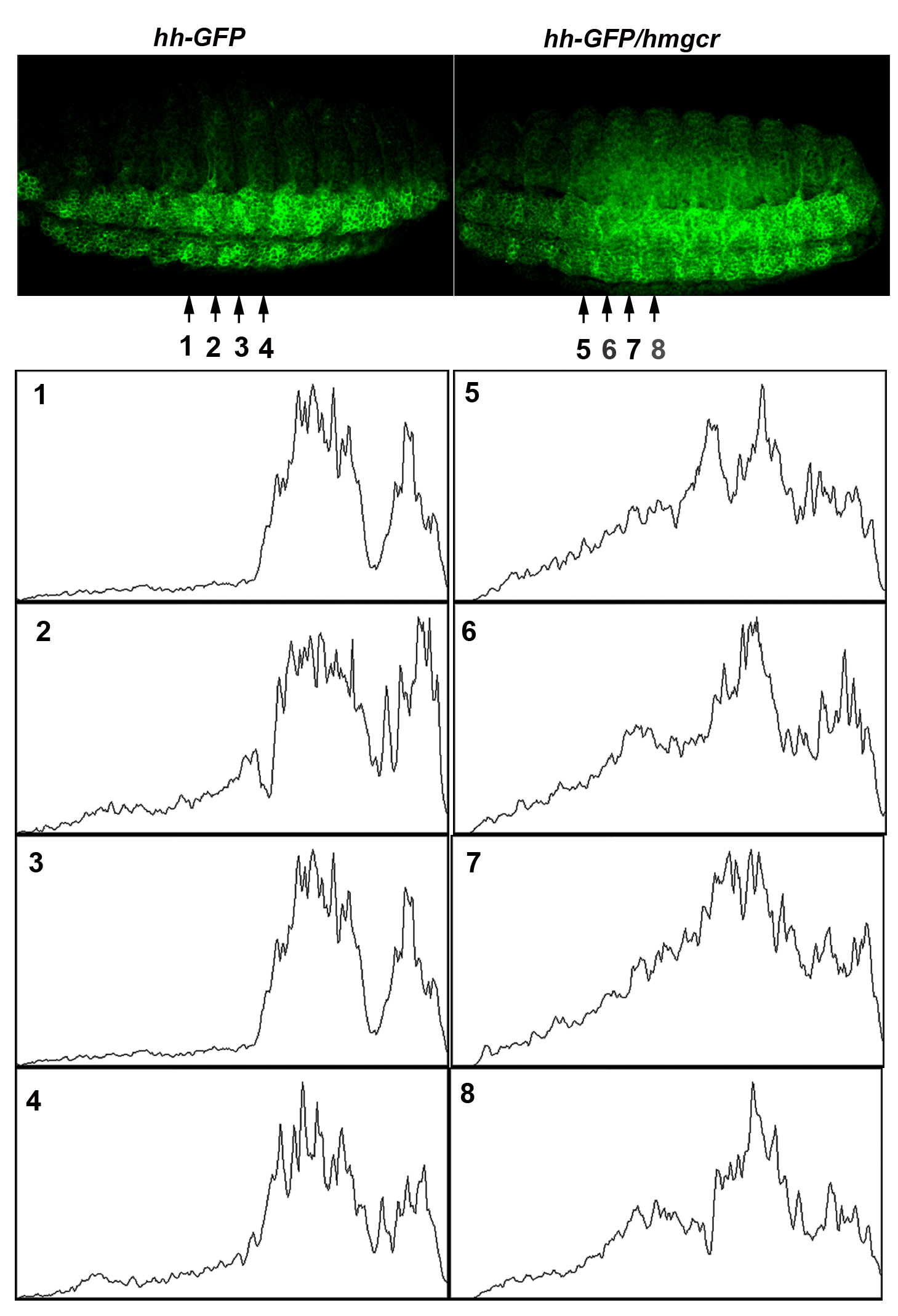

Supplement: Figure S4 — Distribution of Hh-GFP in stage14 hh-GFP and hh-GFP/hmgcr embryos. Embryos in Figure S4 are from panels C and D of Figure 8. This supplemental figure shows a plot of the pixel density (using ImageJ) in an approximately 3 cell wide vertical stripe. The center of each of these stripes is indicated by the arrows below each embryo. There are four plots for each embryo. (TIF) [file pgen.1003720.s004.tif]

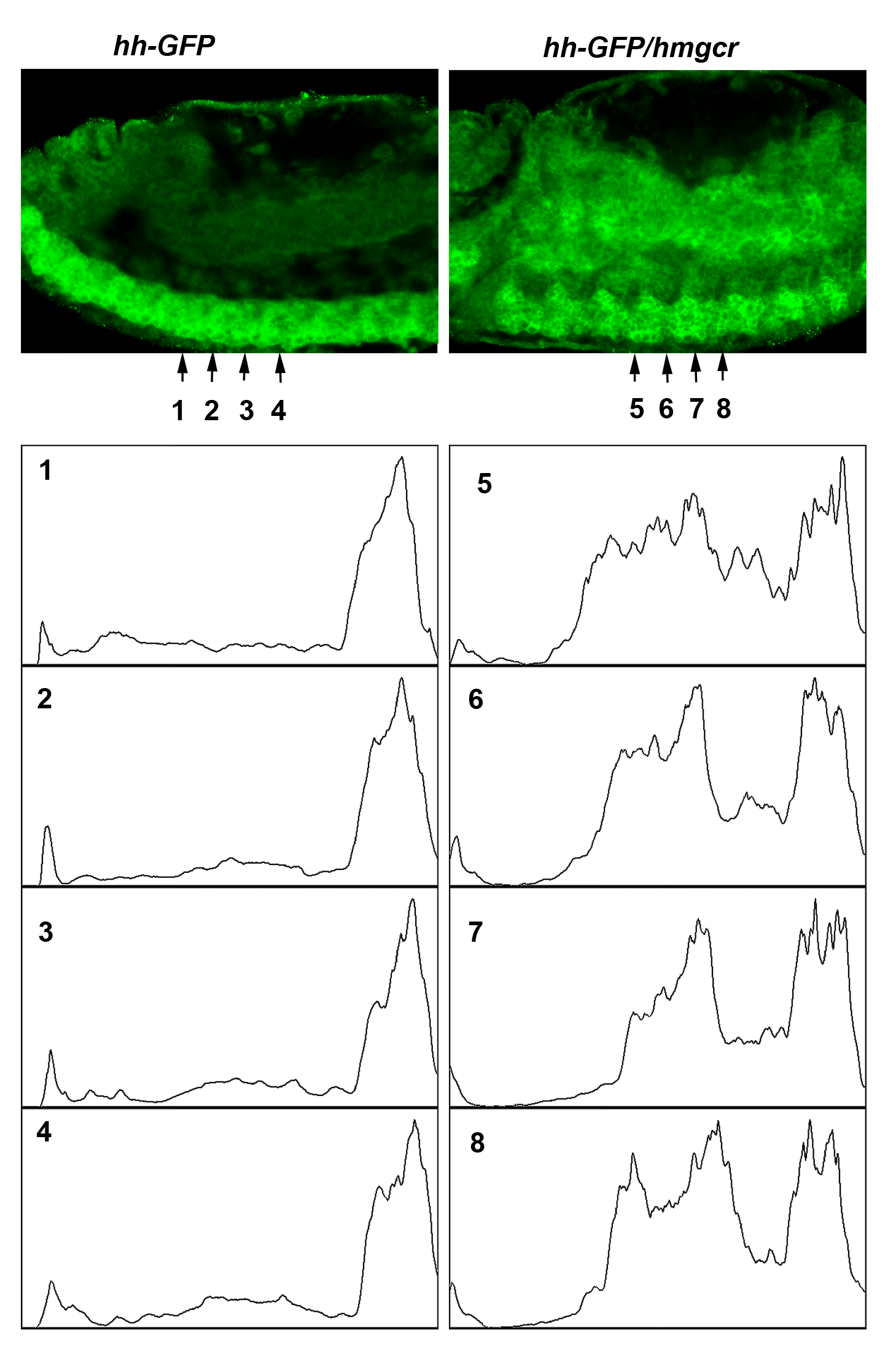

Supplement: Figure S5 — Distribution of Hh-GFP in stage 14 hh-GFP and hh-GFP/hmgcr embryos. Embryos in Figure S5 are from panels E and F of Figure 8. This supplemental figure has a plot of the pixel density (using ImageJ) in an approximately 3 cell wide vertical stripe. The center of each of these stripes is indicated by the arrows below each embryo. There are four plots for each embryo. (TIF) [file pgen.1003720.s005.tif]

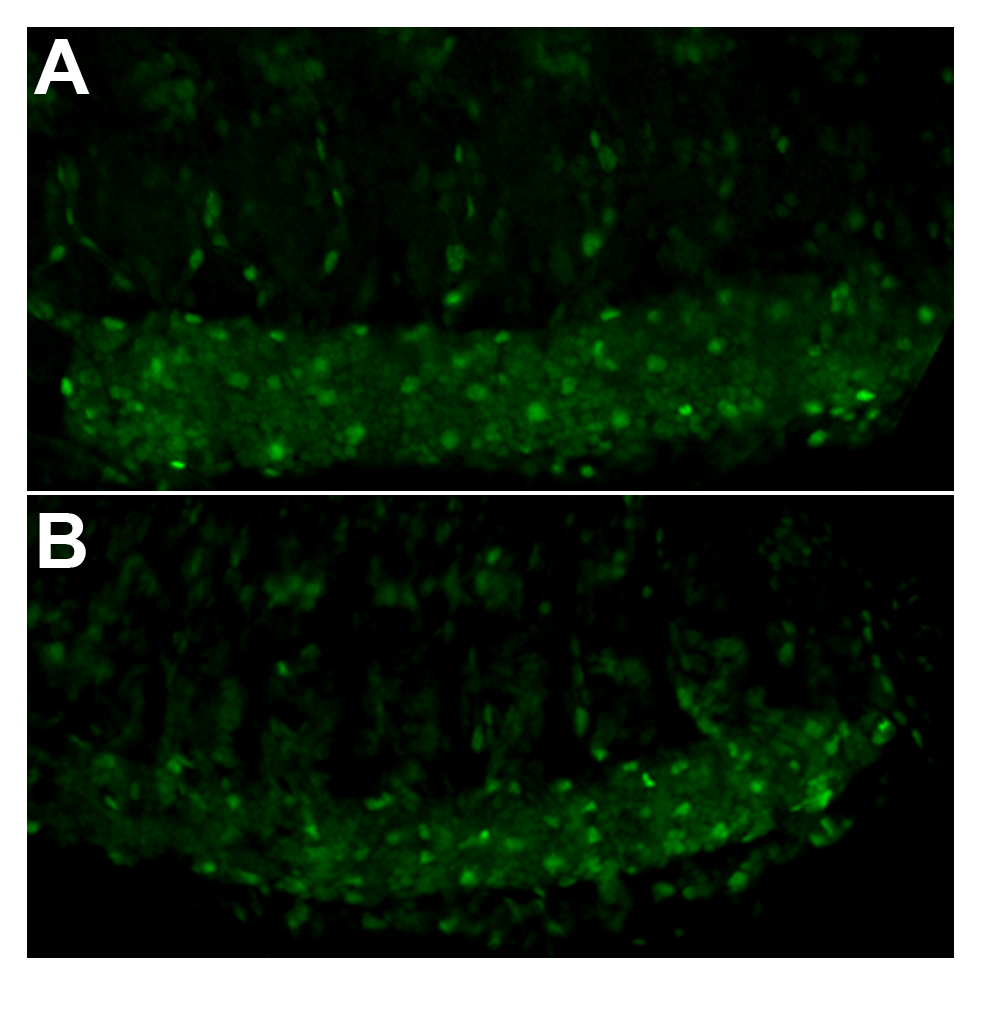

Supplement: Figure S6 — The 2nd and recombined 3rd chromosome elav-GAL4 transgenes drive equivalent levels of expression of a UAS-GFP(NLS) reporter. Females homozygous for the recombined UAS-hmgcr, elav-GAL4 3rd chromosome or the 2nd chromosome elav-GAL4 driver (elav-GAL4/CyO Actin GFP) were mated to UAS-GFP(NLS) males. The resulting embryos were fixed and stained with anti-GFP antibody. (A) elav-GAL4 (2nd)/UAS-GFP(NLS). (B) UAS-hmgcr,elav-GAL4 (3rd)/UAS-GFP(NLS). The signal intensity in each nuclei was estimated by doing pixel counts over a fixed area using ImageJ software. The average pixel intensity per nucleus for elav-Gal4/UAS-GFP(NLS) was 69.8 (S.D.+/−9.7; n = 15) whereas for UAS-hmgcr,elavGal4 embryos was 66 (S.D.+/−13.5; n = 15). (TIF) [file pgen.1003720.s006.tif]
