# Supplementary material for: The hedgehog Pathway Gene shifted Functions together with the hmgcr-Dependent Isoprenoid Biosynthetic Pathway to Orchestrate Germ Cell Migration
Source: PLoS Genet. 2013 Sep 12;9(9):e1003720. doi: 10.1371/journal.pgen.1003720 (PMC3772052; doi:10.1371/journal.pgen.1003720)
Supplement: Text S1 — Does ectopic Hh perturb germ cell migration? The data in Figures 5, 7 and S2 showing that hh misexpression perturbs PGC migration are contradicted by experiments reported in Figure 2 of a paper “Hedgehog does not guide migrating Drosophila germ cells” that was published in 2009 by Renault et al. [29] in Developmental Biology. The material presented in Text S1 addresses this controversy. (PDF) [file pgen.1003720.s007.pdf]

Text Supplemental 1

In 2009 Andrew Renault, Sara Ricardo, Prabhat S. Kunwa, Ana Santos, Michelle Starz-Gaiano, Jennifer A. Stein and Ruth Lehmann published a paper in *Developmental Biology* [29] entitled *"Hedgehog does not guide migrating Drosophila germ cells"* that called into question several of our previous publications connecting Hh to the isoprenoid biosynthetic pathway (e.g., the *hmgcr→qm→ gγ1*) and to the process of PGC migration. In this supplemental text, we address this controversy.

We do so for two reasons. First, the findings reported in Renault *et al.*, [29] contradict experiments in this paper and would cast doubt on their validity. Second, our failure to explicitly address this more than decade old controversy in a public forum has undermined our credibility in the scientific community, jeopardizing our careers.

Below we have reviewed the history of the controversy and discussed some of the most critical discrepancies between our results and those of Renault *et al.*, [29]. Additional documents relating to the Renault *et al* [29] publication are listed below and are available upon request.

Does ectopic hh perturb PGC migration?

The data in Figures 5, 7 and Figure S2 of this paper showing that *hh* misexpression perturbs PGC migration are directly contradicted by experiments reported in Figure 2 of the "Hedgehog does not guide migrating Drosophila germ cells" publication by Renault *et al.,* 2009 ([29]). In contrast to our findings, Figure 2 of Renault *et al.,* shows that driving *hh* expression in the mesoderm with *twist-GAL4* or in the nervous system with *elav-GAL4* has *absolutely* no effect on PGC migration.

Figure 2 in Renault *et al.,* [29] is, in fact, a composite of two figures (Figure 2 and 3) and a table that were included in an older manuscript titled "*Hedgehog does not guide migrating germ cells. Comments on the article by G. Deshpande, L. Swanhart, P. Chiang and P. Schedl: Hedghog Signaling in Germ Cell Migration. Cell 106: 759-769, 2001.*" This manuscript was written by Ruth Lehmann, Prabhat Kunwar, Ana Santos, Michelle Starz-Gaiano, and Jennifer Stein, and was submitted to *Cell* in February 2002.

The three embryos shown at the top of Figure 2 in Renault *et al.*, [29] are identical to the three embryos that were shown in Figure 3 of the Lehmann *et al.*, 2002 manuscript. The data in the Excel graph at the bottom of Figure 2 in Renault *et al.*, [29] are the same as the data that was presented in Figure 2 of Lehmann *et al.*, 2002 (although the labeling and color schemes for the 4 classes of embryos have been updated in the 2009 publication). The table in Lehmann *et al.*, 2002 was part of the legend to Figure 2 and it gives the actual number of embryos in each class for the different genotypes in the bar graph. This table is not included in Renault *et al.*, [29] but is reproduced below (Table 1). The total number of embryos listed for each genotype in the Lehmann *et al.*, 2002 table are identical to the number of embryos listed for the same genotype beneath the Excel graph in Renault *et al.*, [29]. The

percentages in each class are also the same in the Excel 2002 (see reproduction in Figure 1 below) and the Excel 2009 (see Figure 2 in [29]) graphs.

After the "Comments on…" manuscript had been reviewed and recommended for publication by the referees, Dr. V. Siegel, who was editor of *Cell* at the time, contacted us and asked if we wished to retract the Deshpande *et al.*, 2001 [8] paper referred to in the title of the Lehmann *et al*. manuscript. Since we had learned about the contents of the "Comments on…" manuscript in January 2002 from Dr. Lehmann, and had already begun repeating several of the Deshpande *et al.,* [8] experiments, we informed Dr. Siegel that we would make no formal comment until we had finished these experiments.

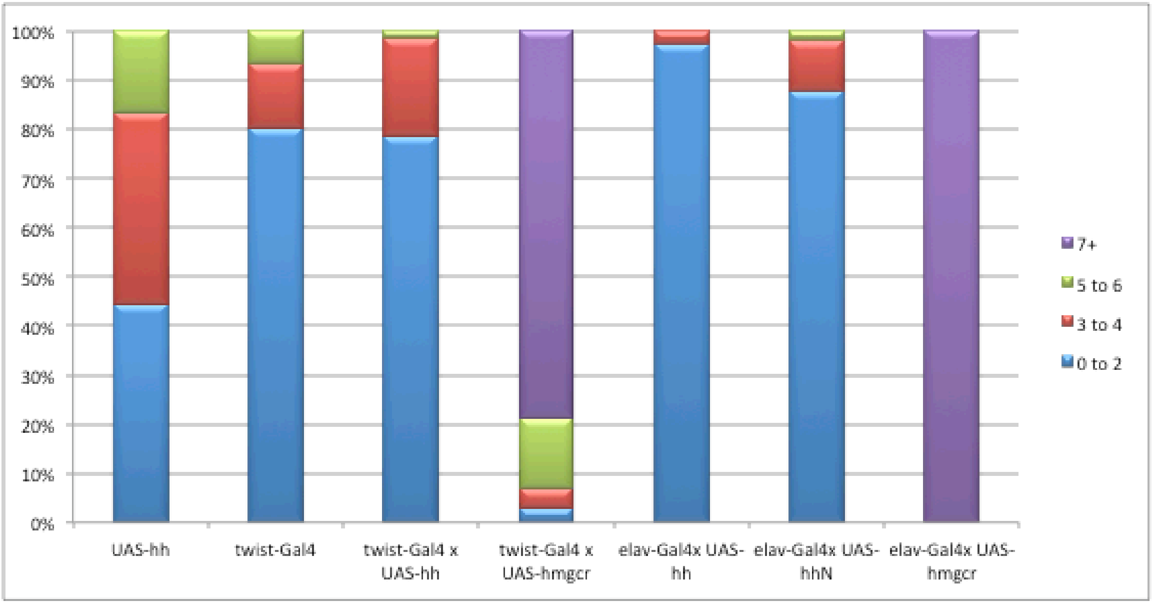

Figure 1. Effects of different *UAS/GAL4* combinations on PGC migration. Data used in the graph are from the table in the legend to Figure 2 of the Lehmann *et al.,* 2002 manuscript.

While there were a number of discrepancies between our publication and the "Comments on…" manuscript that could have been explained by differences in procedures, conditions, genotypes, or some type of error, this was not true for the experiments that documented the effects of ectopic *hh* on PGC migration. These are simple experiments with no possibility for inadvertent or foolish mistakes. Moreover, the discrepancies between our results and those in Lehmann *et al.,* 2002 were so large that they couldn't readily be explained by differences in experimental conditions or methods of analysis. The clear implication of the "Comments on…" manuscript was that the *hh* ectopic expression results in Deshpande *et al.,* [8] were fabrications. This meant that even if we reproduced our previous findings, this would not be sufficient to answer the critique of our *Cell* paper in the Lehmann *et al.,* manuscript because there would be serious doubts about our scientific integrity. For this reason we had already asked Dr. DiNardo at U. Penn to repeat the *hh* ectopic expression experiments even before Dr. Siegel contacted us. When we told Dr. Siegel that Dr. DiNardo had agreed to act as an independent arbitrator, she insisted that he communicate only with *Cell*, and not with us.

Summary of *UAS-hh* misexpression experiments (see figure 2)

Number of PGCs lost	*UAS-hh*	*twist-GAL4*	*twist-GAL4 UAS-hh*	*twist-GAL4 x UAS-hmgcr*	*elav-GAL4x UAS-hh*	*elav-GAL4x UAS-hhN*	*elav-GAL4x UAS-hmgcr*
0 to 2	24	49	48	2	34	43	0
3 to 4	21	8	12	3	1	5	0
5 to 6	9	4	1	10	0	1	0
7 +	0	0	0	56	0	0	51
Total number of embryos	54	61	61	71	35	49	51

Table 1 Embryo counts for each genotype. Table adapted from the table in the legend to Figure 2 of Lehmann *et al.,* 2002.

Dr. DiNardo sent Dr. Siegel his initial report and subsequently an updated report with some additional data towards the end of March 2002. In his report, Dr. DiNardo indicated that he had confirmed our published findings, and *not* those presented in the Lehmann *et al.,* manuscript. His results are presented in Figure 2 below. We received a copy of Dr. DiNardo's updated report from Dr. Siegel at the end of March. Since we had by then finished redoing many of the experiments in Deshpande *et al*., we sent Dr. Siegel a detailed report describing the results of these experiments and responding to the other allegations about our *Cell* publication that were raised in the Lehmann *et al.,* 2002 "Comments on…" manuscript.

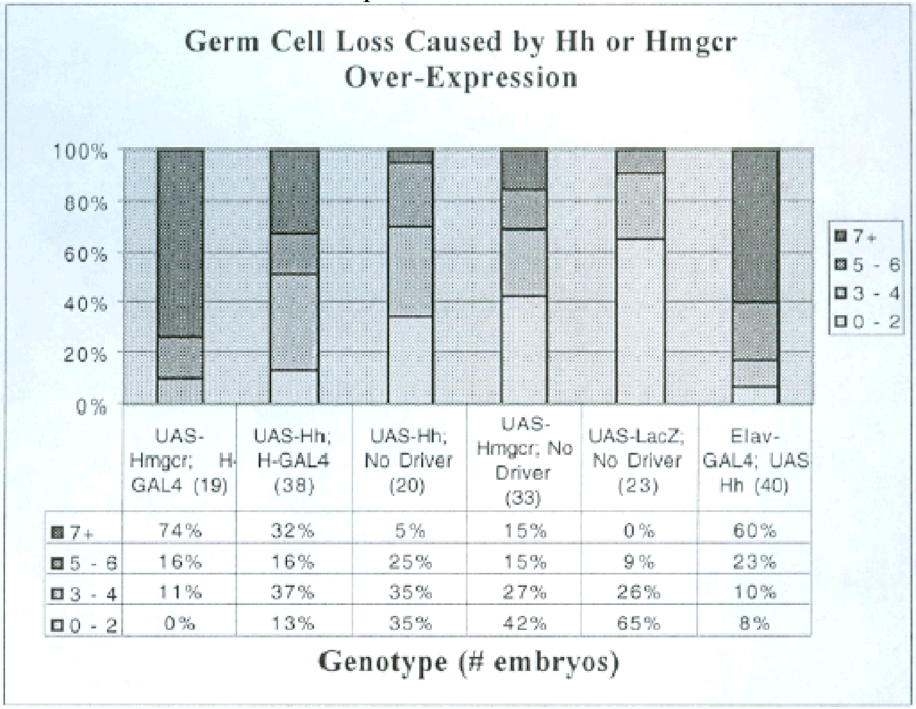

Figure 2 Figure taken from Dr. DiNardo's March 2000 report to the editors of *Cell.*

Because of time pressure, Dr. DiNardo's crosses between *UAS-hmgcr* or *UAS-hh* and *hairy-GAL4* were done by mating homozygous *UAS* transgene females to a mixture of homozygous *hairy-GAL4* and hemizygous *hairy-GAL4/TM3* males. In each case, there will be a mixed population of embryos, some having a single copy of both the driver and the *UAS* transgene and others having just a single copy of the *UAS* transgene. For the experiment with the *elav* driver, males carrying an X linked *elav-GAL4* transgene were mated to females homozygous for the *UAS-hh* transgene. In this case, half of the embryos should have a single copy of both driver and *UAS* transgene, while the other half will have only a single copy of the *UAS* transgene. Thus, Dr. DiNardo's experiments will underestimate the effects of driver dependent *hmgcr* and *hh* expression. When we repeated the ectopic expression experiments, we used males that were hemizgous for the *GAL4* driver or carried an X-linked driver and consequently only half of the embryos in our experiments are expected to carry both the driver and the *UAS* transgene. By contrast, according to Lehmann *et al.,* the parents used in their crosses were in all cases homozygous for the driver and the *UAS* transgene and all progeny should have a single copy of each of the transgenes.

	**DiNardo**	**Lehmann et al., 2002 Figure2**	**Deshpande repeat**	**DiNardo**	**Deshpande repeat**	**Lehmann et al., 2002 Figure2**	**Deshpande repeat**
	elav-GAL4	*elav-GAL4*	*elav-GAL4*	*hairy-GAL4*	*hairy-GAL4*	*twi-GAL4*	*twi-GAL4*
**# lost GC**	(40 embryos)	(35 embryos)	(90 embryos)	(38 embryos	(307 embryo	(61 embryos)	(133 embryo
**7+**	60%	0%	27%	32%	20%	0%	22%
**5-6**	23%	0%	24%	16%	23%	2%	20%
**3-4**	10%	3%	18%	37%	21%	21%	23%
**0-2**	8%	97%	31%	13%	35%	79%	36%

Table 2 Tabulation of migration data from DiNardo, 2002, Deshpande/Schedl, 2002 and Lehmann *et al.,* 2002. Note that the data presented graphically in Figure 2 of Renault *et al.*, [29] are the same as in Lehmann *et al.,* 2002. A subset of the results are presented in this table along with results from Dr. DiNardo's and our reports to *Cell*. For our *hairy-GAL4* experiments two independent drivers were used. The results for one of these (a sum of two experiments) is presented here. Similar results were obtained for the 2nd *hairy-GAL4* driver: see our report to *Cell*.

Table 2 shows a side-by-side comparison of the results from the three sets of experiments. In the case of the *elav* driver it is possible to make a three-way comparison. The results couldn't be more different. Whereas Lehmann *et al.,* 2002 (and [29]) reported that 97% of the *UAS-hh/elav-GAL4* embryos resembled wild type and had only 0-2 lost germ cells, Dr. DiNardo found that less than 10% of the embryos he examined fell into this category. Instead, he found that about 80% of the *UAS-hh/elav-GAL4* embryos had 5 or more lost germ cells. Lehmann *et al.,* 2002, by contrast, didn't detect any embryos that were in this category. Dr. DiNardo also tested the effects of expressing *hh* using the *hairy-GAL4* driver. As was the case for the *elav* driver, he got results completely consistent with those we reported in Deshpande *et al.,* [8]. We also repeated our ectopic expression experiments

using the *elav*, *hairy* and *twist* drivers and as indicated in Table 2 we were able to reproduce the findings we had reported in our 2001 *Cell* publication.

Though Dr. DiNardo's and our experiments were imperfect (because many embryos won't have both the driver and the *UAS* transgene), it is important to emphasize that the effects of ectopic *hh* on PGC migration were in all cases *clear-cut and completely unambiguous*. Moreover, the stark discrepancies between DiNardo's/our findings and the results of the *hh* ectopic expression experiments presented in Lehmann *et al.,* 2002 (or Renault et al., [29]) aren't readily explained by differences in scoring criteria or experimental conditions as all three groups obtained similar results for the positive and negative controls.

Does patchedΔloop2 expression in PGCs induce migration defects?

The Renault *et al*., [29] *Developmental Biology* paper also questioned experiments in Deshpande *et al.,* [8] showing that components of the machinery involved in receiving and responding to Hh are needed in PGCs for proper migration. Many of the same questions and allegations were, in fact, raised in the original Lehmann *et al*., 2002 "Comments on…" manuscript and were addressed at that time in the report we sent to *Cell*. This report is included in the documents available on request.

Not addressed in our report to *Cell* was an experiment in Lehmann *et al*., 2002 which purportedly showed that ectopic expression of a dominant negative *patched* protein, *patchedΔloop2*, in PGCs had no effect on their migration. Precisely this same 2002 experiment appears in Figure 3 of Renault *et al*., [29]. In this case it was used to call into question a *patchedΔloop2* experiment we (Deshpande, Sethi, and Schedl) did several years after the original Lehmann *et al.,* manuscript and published in *Genetics* in 2007 [44].

In the Deshpande *et al.,* [44] experiment we examined the effects of expressing *patchedΔloop2* in the germline on PGC migration. For this purpose we crossed males carrying an X-linked *UAS: patchedΔloop2* transgene (from Dr. G. Struhl) to females homozygous for a *nos-GAL4* driver. Since the *UAS* transgene is X-linked only female embryos will carry both the *GAL4* driver and the *UAS* transgene. For this reason, we sexed the embryos using Sex-lethal antibody and then assessed PGC migration. As indicated in Table 3 below, 100 female embryos were examined and about 30% exhibited noticeable PGC migration defects. By contrast, PGC migration in males carrying only the *GAL4* driver resembled wild type.

	**Renault *et al.* [29]**		**Deshpande *et al.* 2007**	
	"ptcΔloop2" ***nos-GAL4***		*nos-GAL4*	*ptcΔloop2* *nos-GAL4*
**# lost GC**	14 embryos sex unknown		122 male embryos	100 female embryos
**7+**	0%	**7+**	2%	14%
**5-6**	0%	**5-6**	6%	17%
**3-4**	14% (2)			
**0-2**	86% (12)	**0-4**	91%	69%

Table 3. Ectopic expression of *patchedΔloop2* in PGCs.

The *patchedΔloop2* experiment in Lehmann *et al*., 2002 was included in Figures 4 and 5. Figure 4 showed a photograph of a "*nos-GAL4xUAS-patchedΔloop2*" embryo with a completely normal looking gonad, while Figure 5 presented an Execl graph of their results. The table in the legend to Figure 5 indicated that the authors examined a total of 14 embryos, of which only 2 showed more than 2 lost PGCs. Though not stated anywhere in the Lehmann *et al*., 2002 manuscript, this *UAS* transgene is on the X chromosome and is, in fact, the same one from Dr. Struhl that we used in our 2007 publication. As in our experiment, only one half of the embryos (females) will have both the UAS transgene and the *nos-GAL4* driver. However, this fact was not stated in the manuscript. Nor was there any indication that the authors determined the sex of the embryos so that they would know which were the experimental and which were the controls. Instead, the legend states that females homozygous for the *nos-GAL4* transgene were mated to males homozygous for the *UAS* transgene "…such that all embryos should express the *UAS* transgene in the germ cells." Accordingly, the percentages in the *patchedΔloop2* graph in Figure 5 were calculated as if *all* embryos had both transgenes and the photo of the embryo with a normal looking embryonic gonad in Figure 4 was labeled "*nos-GAL4xUAS-patchedΔloop2*" as if its genotype had been unambiguously established.

Figure 3 of Renault *et al*., [29] has the same data as was shown in Figures 4 and 5 of Lehmann *et al.,* 2002 including this particular *patchedΔloop2* experiment. In panel C of Figure 3 is a photo of the same "*nos-GAL4xUAS-patchedΔloop2*" embryo that was presented in Figure 4 of Lehmann *et al*., 2002. Also as in the original *Cell* manuscript, 14 embryos were examined and the calculation presented in the Excel graph in Figure 3 assumes that *all* 14 embryos carry both the *UAS-patchedΔloop2* transgene and the *nos-GAL4* driver. There is no indication in either the figure legend or in the text in the results section describing the experiment in Figure 3 that only about half of the 14 embryos are expected to have both the *UAS-patchedΔloop2* transgene and the *nos-GAL4* driver. Nor is there any indication that the genotype of the embryo in panel C that is labeled "*nos-GAL4xUAS-patchedΔloop2*" and has a normal looking gonad is actually unknown. On the other hand, in contrast to *Cell* text, the Materials and Methods section in Renault *et al*., [29] states that in this particular experiment only female embryos will have both transgenes.

Documents available on request:

a) Lehmann et al., 2002 manuscript.
b) DiNardo report to *Cell.*
c) Our report to *Cell.*

References:

44. Deshpande G, Sethi N, Schedl P (2007). *toutvelu*, a regulator of heparan sulfate proteoglycan biosynthesis, controls germ cell migration. Genetics 176: 905-12.
